# Supplementary material for: The stress-induced transcription factor ATF4 has multiple conserved retrocopies that can alter gene expression
Source: HGG Adv. 2026 Aug 27;7(4):100666. doi: 10.1016/j.xhgg.2026.100666 (PMC13587735; doi:10.1016/j.xhgg.2026.100666)
Supplement: Supplementary file 1 [file mmc1.pdf]

HGGA, Volume 7

## Supplemental information

# The stress-induced transcription factor ATF4 has multiple conserved retrocopies that can alter gene expression

**Hans M. Dalton, Elizabeth M. Brydon, Tiffany S. Chan, Katie G. Owings, Mandi M.L. Wild, Naomi J. Young, and Clement Y. Chow**

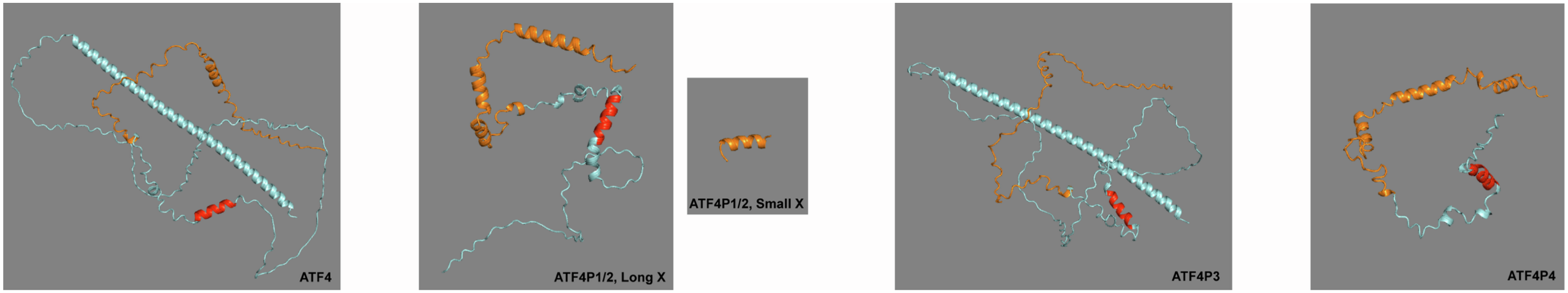


**Figure S1. AlphaFold predictions of ATF4 retrocopy protein structures.** Alphafold 3 predictions of translated retrocopy transcripts, visualized in PyMOL. Specific protein domains are colored for comparison: the p300 interaction domain (orange), an alpha helix structure that is maintained in all proteins except ATF4P1/2 Small X (red), and the rest of the protein including the basic leucine zipper domain (blue

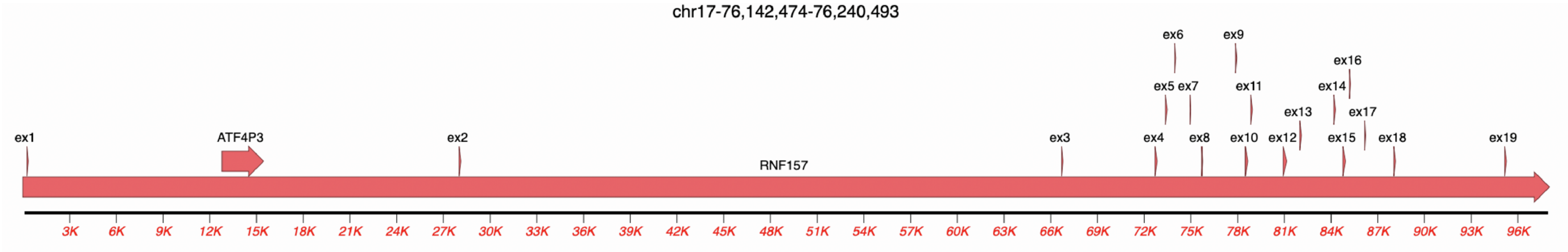


**Figure S2. Mock-up of the genomic region containing the gene *RNF157*.** *ATF4P3* was inserted between exons 1 and 2. Image was created using MacVector™ v.18.2.5.

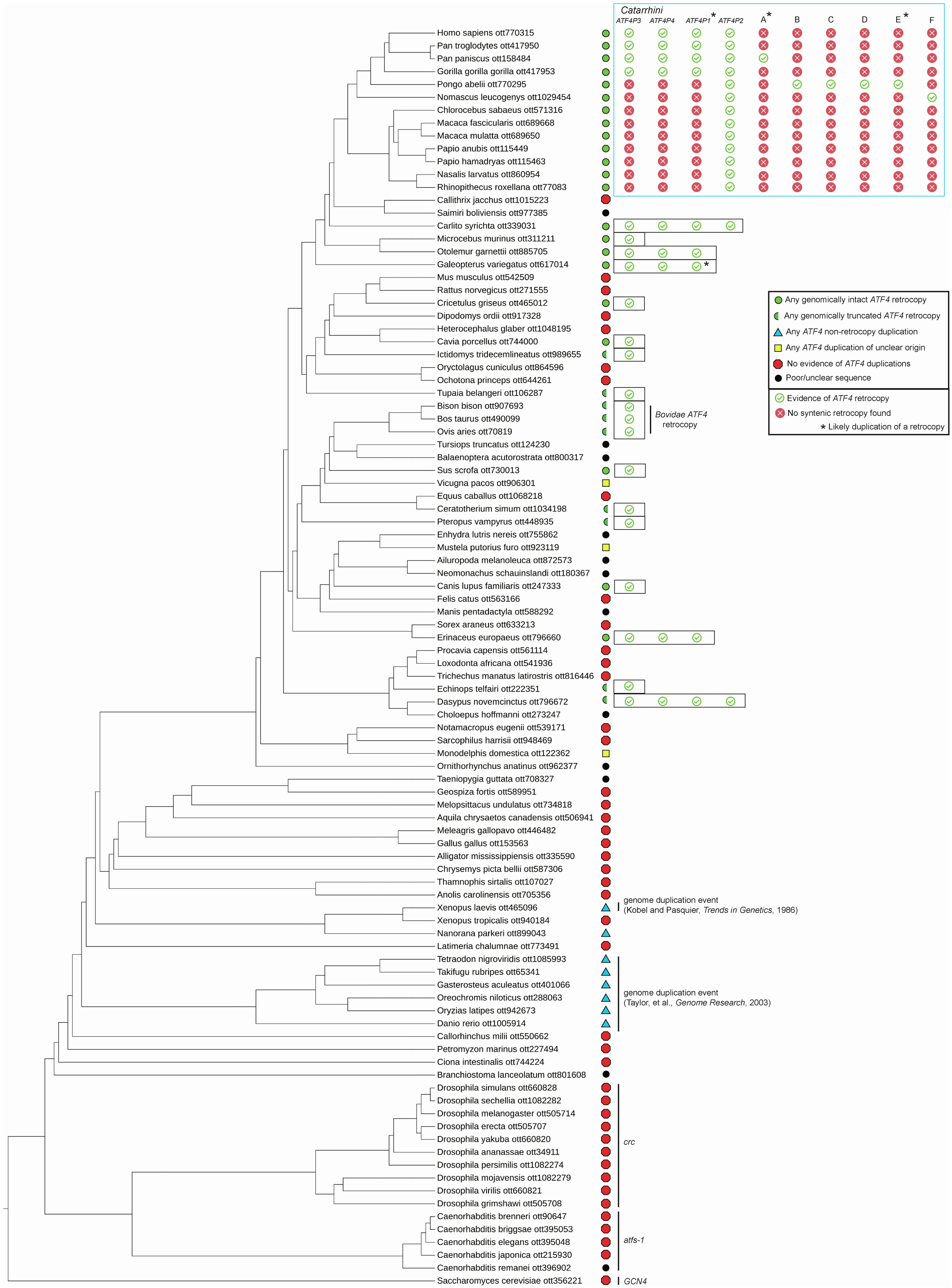

Catarrhini
ATF4P3
ATF4P4
ATF4P1 *
ATF4P2
A *
B
C
D
E *
F
Homo sapiens ott770315
Pan troglodytes ott417950
Pan paniscus ott158484
Gorilla gorilla gorilla ott417953
Pongo abelii ott770295
Nomascus leucogenys ott1029454
Chlorocebus sabaeus ott571316
Macaca fascicularis ott689668
Macaca mulatta ott689650
Papio anubis ott115449
Papio hamadryas ott115463
Nasalis larvatus ott860954
Rhinopithecus roxellana ott77083
Callithrix jacchus ott1015223
Saimiri boliviensis ott977385
Carlito syrichta ott339031
Microcebus murinus ott311211
Otolemur garnettii ott885705
Galeopterus variegatus ott617014
Mus musculus ott542509
Rattus norvegicus ott271555
Cricetulus griseus ott465012
Dipodomys ordii ott917328
Heterocephalus glaber ott1048195
Cavia porcellus ott744000
Ictidomys tridecemlineatus ott989655
Oryctolagus cuniculus ott864596
Ochotona princeps ott644261
Tupaia belangeri ott106287
Bison bison ott907693
Bos taurus ott490099
Ovis aries ott70819
Bovidae ATF4 retrocopy
Tursiops truncatus ott124230
Balaenoptera acutorostrata ott800317
Sus scrofa ott730013
Vicugna pacos ott906301
Equus caballus ott1068218
Ceratotherium simum ott1034198
Pteropus vampyrus ott448935
Enhydra lutris nereis ott755862
Mustela putorius furo ott923119
Ailuropoda melanoleuca ott872573
Neomonachus schauinslandi ott180367
Canis lupus familiaris ott247333
Felis catus ott563166
Manis pentadactyla ott588292
Sorex araneus ott633213
Erinaceus europaeus ott796660
Procavia capensis ott561114
Loxodonta africana ott541936
Trichechus manatus latirostris ott816446
Echinops telfairi ott222351
Dasypus novemcinctus ott796672
Choloepus hoffmanni ott273247
Notamacropus eugenii ott539171
Sarcophilus harrisii ott948469
Monodelphis domestica ott122362
Ornithorhynchus anatinus ott962377
Taeniopygia guttata ott708327
Geospiza fortis ott589951
Melopsittacus undulatus ott734818
Aquila chrysaetos canadensis ott506941
Meleagris gallopavo ott446482
Gallus gallus ott153563
Alligator mississippiensis ott335590
Chrysemys picta bellii ott587306
Thamnophis sirtalis ott107027
Anolis carolinensis ott705356
Xenopus laevis ott465096
Xenopus tropicalis ott940184
Nanorana parkeri ott899043
Latimeria chalumnae ott773491
genome duplication event
(Kobel and Pasquier, Trends in Genetics, 1986)
Tetraodon nigroviridis ott1085993
Takifugu rubripes ott65341
Gasterosteus aculeatus ott401066
Oreochromis niloticus ott288063
Oryzias latipes ott942673
Danio rerio ott1005914
genome duplication event
(Taylor, et al., Genome Research, 2003)
Callorhinchus milii ott550662
Petromyzon marinus ott227494
Ciona intestinalis ott744224
Branchiostoma lanceolatum ott801608
Drosophila simulans ott660828
Drosophila sechellia ott1082282
Drosophila melanogaster ott505714
Drosophila erecta ott505707
Drosophila yakuba ott660820
Drosophila ananassae ott34911
Drosophila persimilis ott1082274
Drosophila mojavensis ott1082279
Drosophila virilis ott660821
Drosophila grimshawi ott505708
crc
Caenorhabditis brenneri ott90647
Caenorhabditis briggsae ott395053
Caenorhabditis elegans ott395048
Caenorhabditis japonica ott215930
Caenorhabditis remanei ott396902
atfs-1
Saccharomyces cerevisiae ott356221
GCN4
Any genomically intact ATF4 retrocopy
Any genomically truncated ATF4 retrocopy
Any ATF4 non-retrocopy duplication
Any ATF4 duplication of unclear origin
No evidence of ATF4 duplications
Poor/unclear sequence
Evidence of ATF4 retrocopy
No syntenic retrocopy found
* Likely duplication of a retrocopy

**Figure S3. Species tree of all organisms that we queried for *ATF4* retrocopies.** Various colored shapes represent the status of any *ATF4* retrocopy and/or duplication found upon searching BLAT (with a BLAT score of at least 100). *Catarrhini* retrocopies, containing all retrocopies of *ATF4* found in humans, are contained in a blue box. In this box, checkmarks represent the presence of an *ATF4* retrocopy being found syntenic to *ATF4P1-P4*, or independent *ATF4* retrocopies that are neither syntenic nor duplications of *ATF4P1-P4* (letters A-F). Outside of this box, each horizontal box represents independent retrocopies of *ATF4* (not related to *ATF4P1-4*), while the single vertical box (labeled *Bovidae* retrocopy) is another syntenic retrocopy of *ATF4*. For example, for *Carlito syrichta*, it has four independent retrocopies unique to itself (i.e. these are not *ATF4P1-4* and are not syntenic to any other primate *ATF4* retrocopy). We also note two cases where entire genomes were duplicated (and *ATF4* with them) in *Xenopus*[1] and ray-finned fish[2]. Finally, we note that we searched the listed closest invertebrate homologs in *Drosophila*, *Caenorhabditis*, and *S. cerevisiae*, none of which had retrocopies.

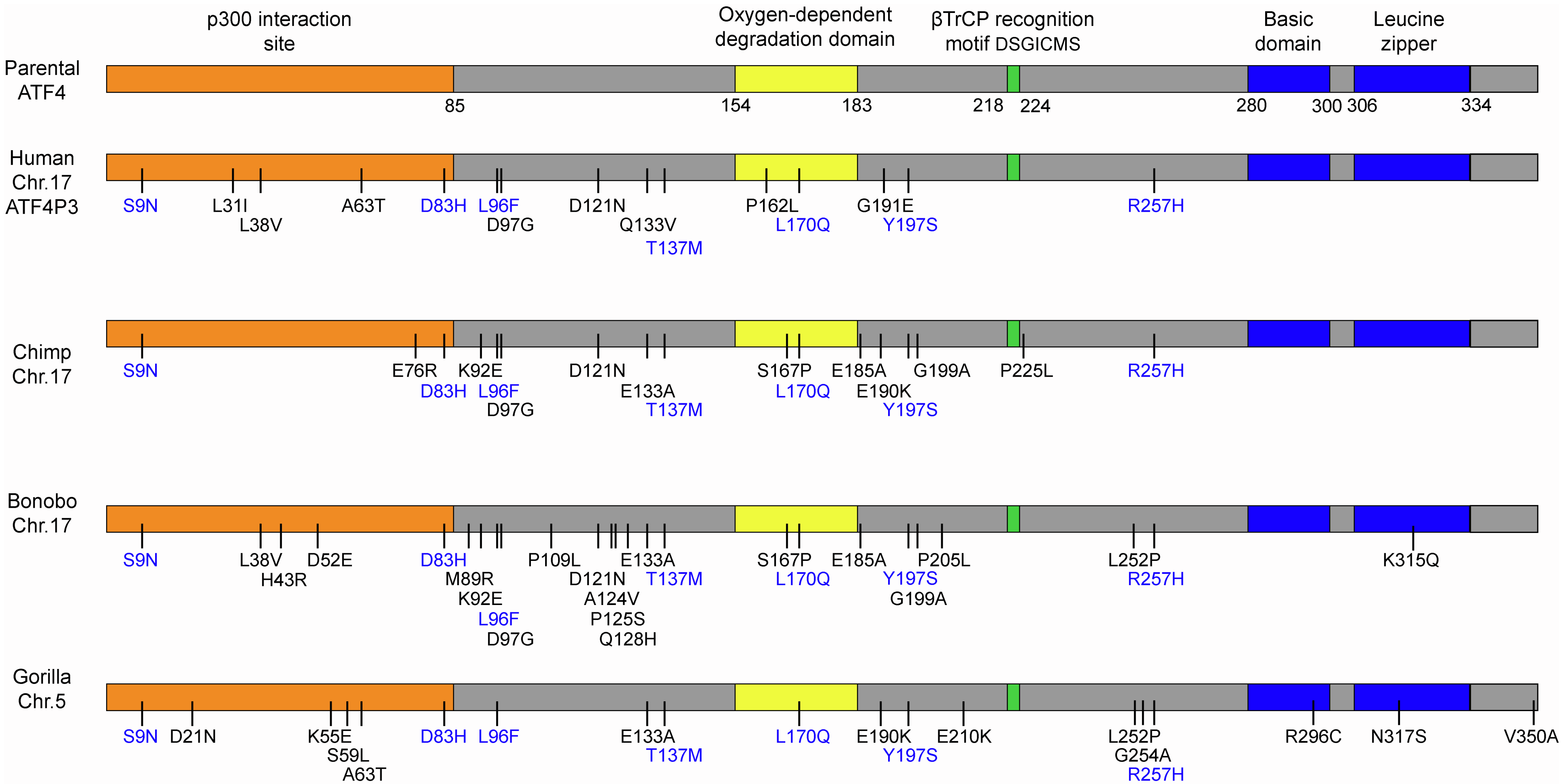


**Figure S4. Mock-up of ATF4P3 across humans, chimps, bonobos, and gorillas.** We compared protein sequences to the species-specific ATF4 parent gene to determine amino acid changes. Amino acid changes that are shared across all four species are labeled in blue.

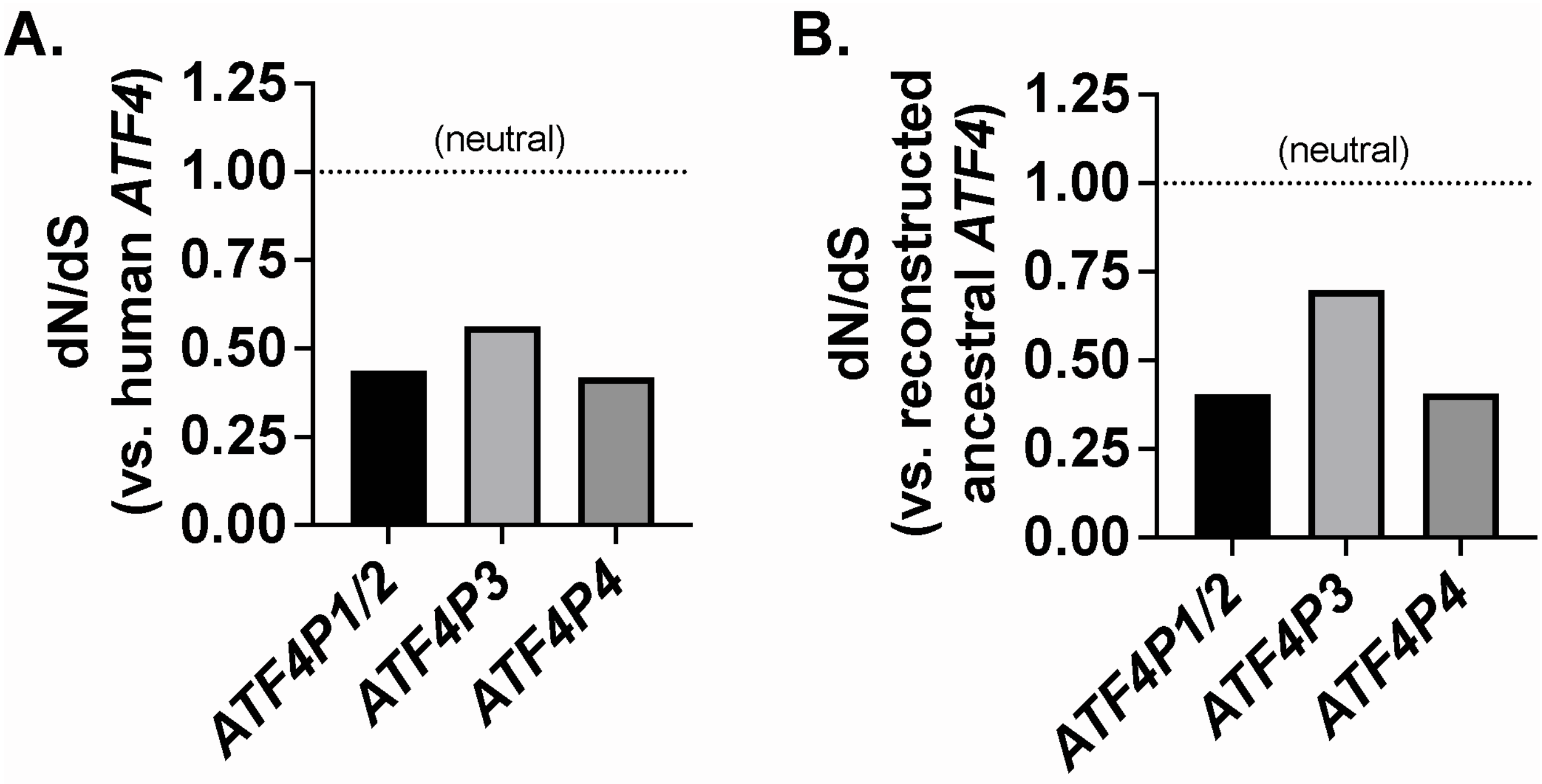


**Figure S5. Graphs of dN/dS calculations on *ATF4* retrocopies.** A. Graph showing calculated dN/dS of *ATF4* retrocopies compared to the human *ATF4* parent gene. B. Graph showing calculated dN/dS of *ATF4* retrocopies compared to the ancestral reconstructions of *ATF4* (Table S3). The dotted line at 1.0 indicates neutral evolution, while below line represents negative selection.

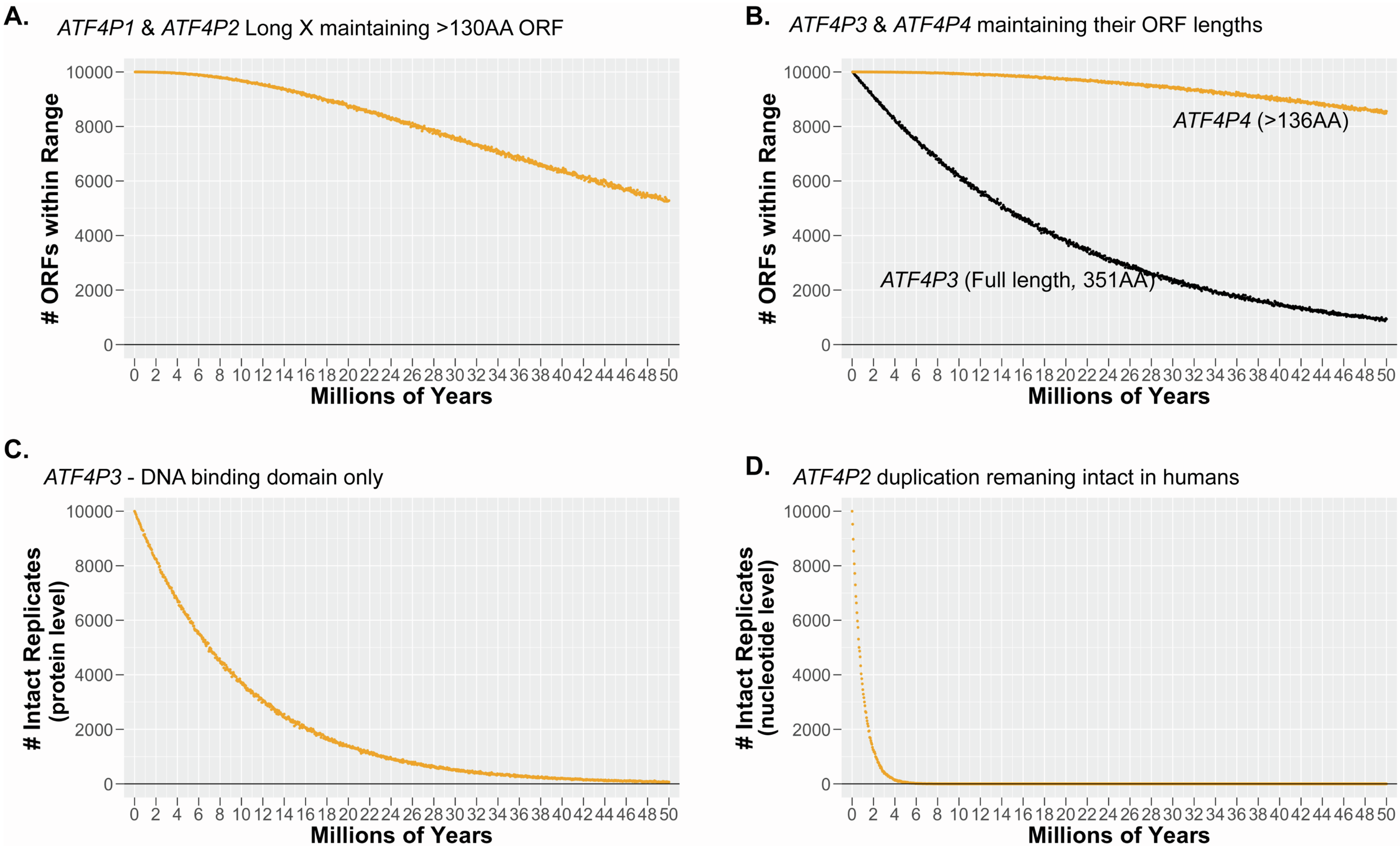


**Figure S6. Graphs of computationally modeled mutation rates of *ATF4* retrocopies.** A. Graph showing how many permutations (out of 10,000) of our estimated ancestral copy of *ATF4P1/P2* maintaining a >130 AA ORF over time. B. Same as (A), but for ATF4P3 and ATF4P4 maintaining either >136 AA ORF (*ATFP4*) or a full length ORF (*ATF4P3*). C. Same as (A), but for ATF4P4 maintaining its DNA binding domain fully intact. D. Same as

(A), but for *ATF4P2* duplication remaining invariant. These graphs were used to determine probabilities of intactness across species - see Methods for further details.

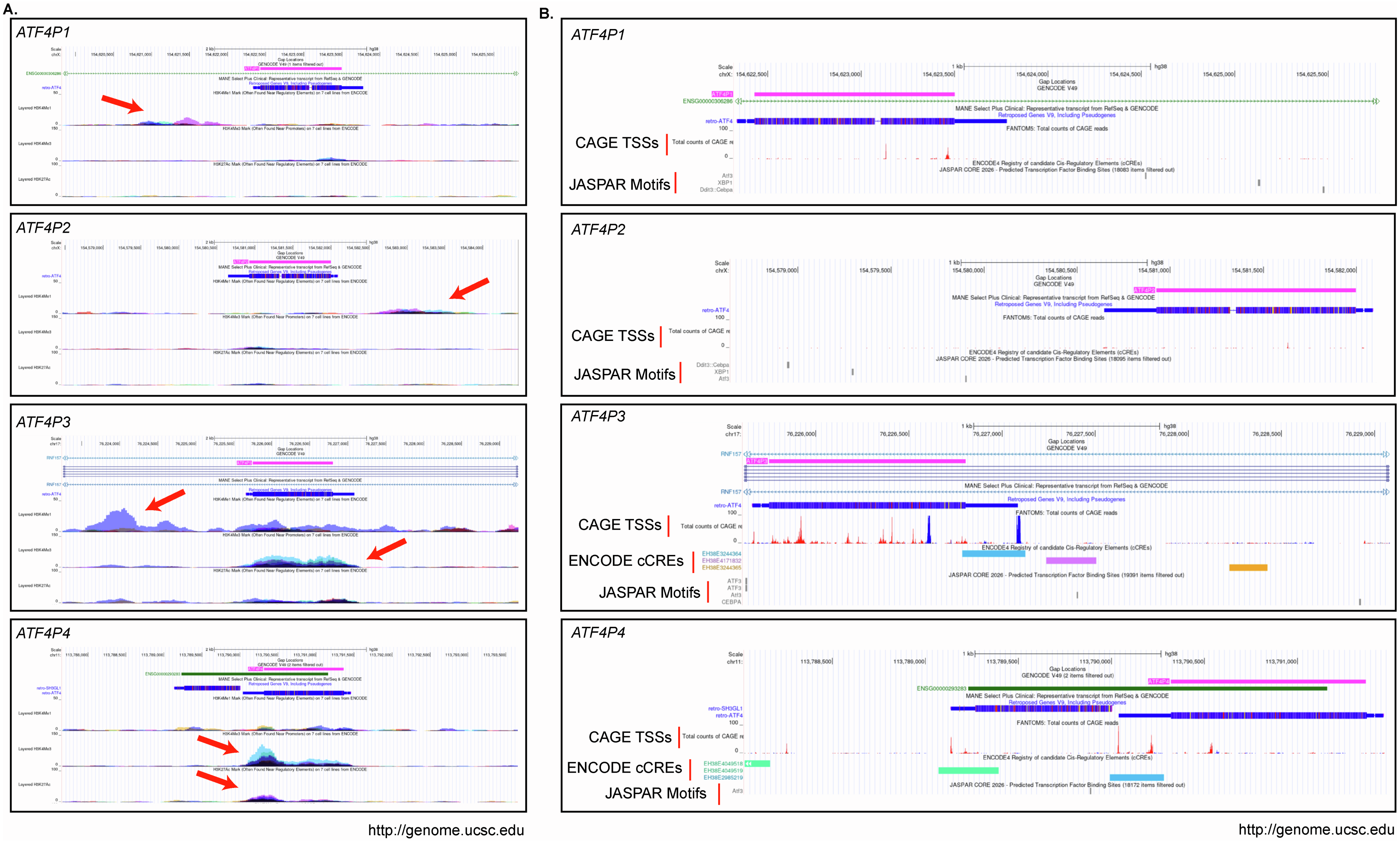


**Figure S7. UCSC genome browser navigated to retrocopies with tracks for regulatory information.** A. Tracks containing the chromatin markers H3K27Ac, H3K4Me1, and H3K4Me3 are shown within a 6 kb window. We point out peaks that are higher than surrounding areas with red arrows. Note that >2 kb on either side display no H3K4Me3 marks, suggesting specificity to each retrocopy. B. Tracks containing cCREs from ENCODE,

transcription start site (TSS) peaks from CAGE data, and any JASPAR motifs for ISR/UPR-specific transcription factors (see Methods) are shown. For cCREs: blue = high chromatin accessibility/CTCF signals, but low H3K4me3/H3K27ac signals; mauve = low chromatin accessibility/chromatin markers/CTCF signals, but are bound by a transcription factor; orange = high chromatin accessibility/H3K27ac signals and within 2 kb of a TSS; and green = high chromatin accessibility, but low H3K4me3/H3K27ac/CTCF signals. Note that the cCRE track is on for *ATF4P1* and *ATF4P2*, but no cCREs are present.

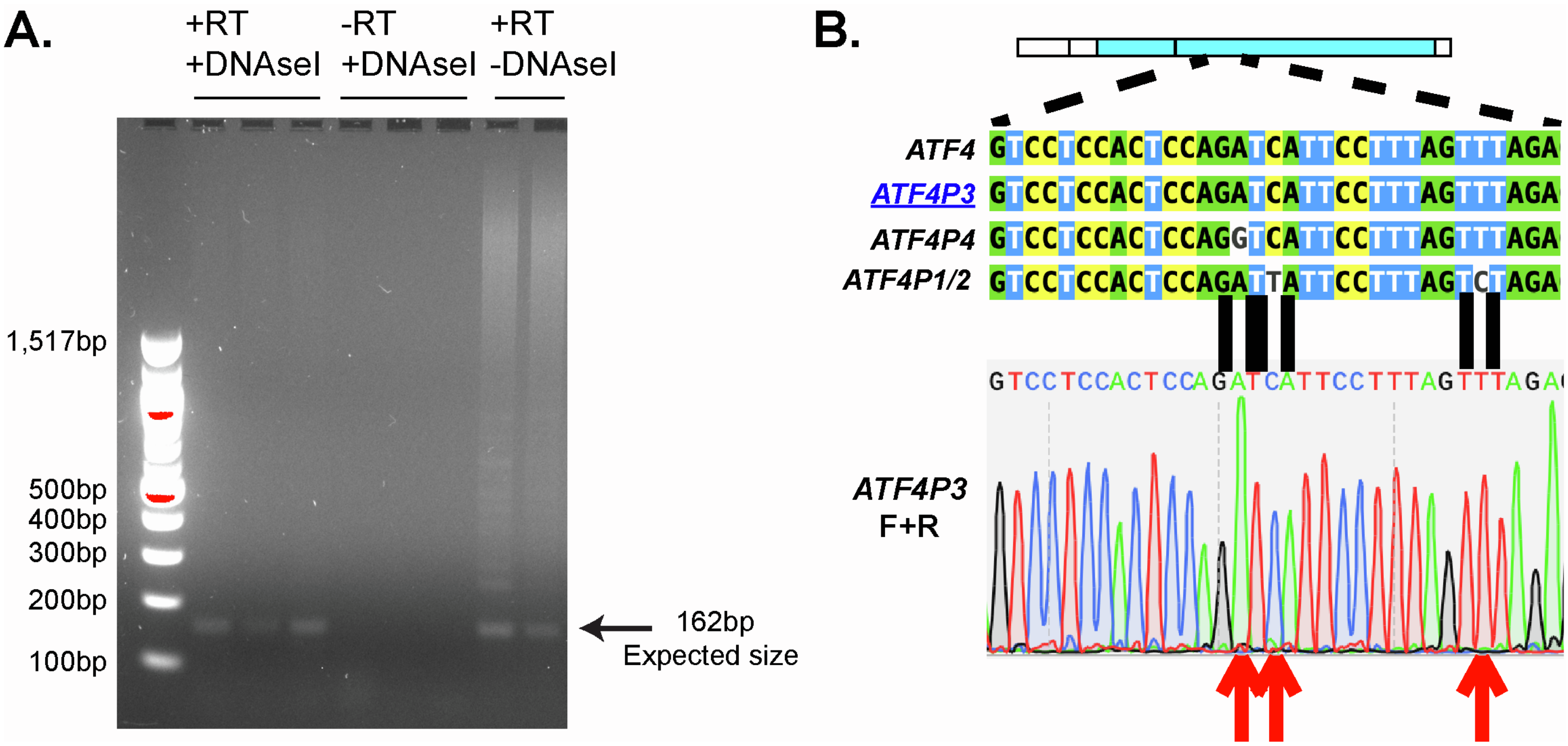


**Figure S8. DNAse efficacy and primer specificity.** A. Agarose gel showing the effectiveness of the Zymo DirectZol™ kit at removing genomic DNA from our TriZol™-derived RNA samples of RPE-1 human cells. We used the *ATF4* Forward and Reverse primers listed for qPCR. Lane 1 is a 100 bp ladder (NEB, cat. N3231L) (the two red splotches represent hitting the light threshold for two sizes that are unrelated to the results). Lanes 2-4 are three biological replicates using the DirectZol kit with the optional DNAseI treatment. Lanes 5-7 are three biological replicates the same procedure but omitting the reverse transcriptase reagent. Lanes 8-9 are biological replicates using the DirectZol kit without the DNAseI treatment. If our prepped RNA sample contained residual genomic DNA, that the DNAseI treatment did not destroy, we would have seen bands in lanes 5-7. Thus, we conclude that the DNAseI treatment does fully remove residual genomic DNA, and that lanes 2-4 (and the data in Figure 3) are derived from actual expressed mRNA. Lanes 8-9 are for reference only to show that DNAse I does have an effect on removing DNA. B. Additional view of chromatogram of sequenced DNA product using ATF4P3 primers. This plot shows three additional SNPs, one for *ATF4P4* and two for *ATF4P1/2*, each of which does not show contamination of the *ATF4P3* transcript.

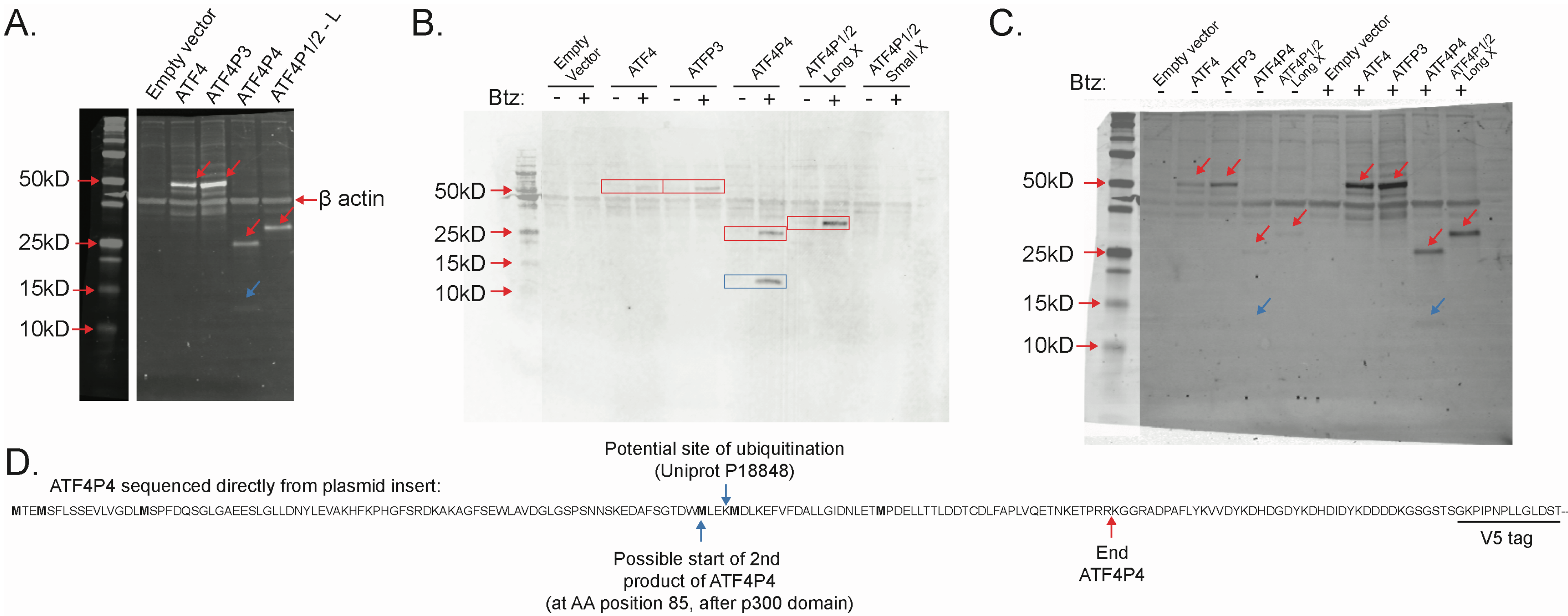


**Figure S9. Images and descriptions of raw Western blots.** A. Western blot of protein extracts from each OE ATF4 cell line using the anti-V5 antibody with no treatment(s). B. Uncropped Western blot of Figure 4. We note here that Btz treatment did not upregulate a visible band for the ATF4P1/2 Small X OE cell line. C. A second biological replicate of a Btz-treatment Western blot, as in (B) and Figure 4. This blot was globally contrast-adjusted to visualize fainter bands. D. Sequenced plasmid insert of ATF4P4 overexpression clone, starting at the ATF4P4 ORF. Note that the ladders are part of the original blot (and are overlaid in their general position on the blot) but are imaged using a different fluorophore, hence the difference in contrast in the ladder.

**Supplemental references.**


1. Kobel, H. R. & Du Pasquier, L. Genetics of polyploid Xenopus. *Trends Genet.* **2**, (1986).
2. Taylor, J. S. *et al.* Genome duplication, a trait shared by 22,000 species of ray-finned fish. *Genome Res.* **13**, (2003).
