# Supplementary material for: The stress-induced transcription factor ATF4 has multiple conserved retrocopies that can alter gene expression
Source: HGG Adv. 2026 Aug 27;7(4):100666. doi: 10.1016/j.xhgg.2026.100666 (PMC13587735; doi:10.1016/j.xhgg.2026.100666)
Supplement: Supplementary file 2 [file mmc7.pdf]

HGG Advances

ARTICLE

The stress-induced transcription factor ATF4 has multiple conserved retrocopies that can alter gene expression

Hans M. Dalton,[1,2,4,*] Elizabeth M. Brydon,[1] Tiffany S. Chan,[2] Katie G. Owings,[1] Mandi M.L. Wild,[2] Naomi J. Young,[1] and Clement Y. Chow[1,3,*]

Summary

The cell must defend against various stressors from internal and external sources that disrupt cell homeostasis. The integrated stress response (ISR) is a highly conserved pathway that helps restore this homeostasis through upregulating the transcription factor ATF4. Despite its importance to cell health and human disease, *ATF4* has several duplications in humans that have not been characterized. Here, we characterize four retroduplications (retrocopies) of *ATF4* in humans. Evolutionary analysis demonstrates that these retrocopies are present and intact in many primate species over the past 37 million years, including several independent copies. We also find evidence of non-neutral evolution among primates. Human *ATF4* retrocopies show basal transcription in healthy, unstressed cells and can be upregulated by the ISR. When translated in human cells, ATF4 retrocopy proteins are regulated by the proteasome in the same way as the parent ATF4 protein. Remarkably, each retrocopy can also alter the expression of several canonical ATF4 target genes, demonstrating that they can impact ISR-ATF4 stress signaling. Overall, *ATF4* retrocopies are conserved, biologically functional, and should be considered in future studies of the ISR and ATF4.

Introduction

Cellular pathways such as the integrated stress response (ISR) are activated in response to stress to restore homeostasis. The ISR is activated by viral infection, amino acid deprivation, endoplasmic reticulum (ER) stress, or heme deprivation through the protein kinases PKR, GCN2, PERK, and HRI, respectively.[1,2] In response to this stress, the ISR inactivates the translation initiation factor eIF2B, downregulating global translation, while simultaneously upregulating specific translation of the ATF4 transcription factor[1,2] (MIM: 604064)[3] (Figure 1A).

ATF4 is basic leucine zipper transcription factor that is critical to restoring cell homeostasis or, under high or prolonged stress, activating apoptosis.[1,4,5] It modulates the expression of hundreds of genes, including those involved in amino acid synthesis, autophagy, and apoptosis.[5–7] ATF4 also has important roles in development[8–11] and tumor biology[8,12–17] in humans. The basic leucine zipper domain allows ATF4 to localize to the nucleus, bind to DNA, and dimerize with proteins such as CHOP and ATF3.[18]

ATF4 is regulated at the protein and mRNA level. ATF4 protein contains a p300 interaction domain that enables interaction with the histone acetyltransferase p300.[19] This interaction increases ATF4 stability and is independent of the p300 acetyltransferase activity.[19] ATF4 also contains two known degradation domains: an oxygen-dependent degradation (ODD) domain and a βTrCP motif.[1] Proline residues in the ODD domain are hydroxylated by the oxygen sensor PHD3 and are then targeted by the ubiquitin ligases Siah1 and Siah2.[20,21] The βTrCP motif can be phosphorylated, leading to targeting by the $SCF^{\beta TrCP}$ ubiquitin ligase.[22–24] Ultimately, each degradation domain leads to ubiquitination and degradation by the proteasome, and, thus, ATF4 has a half-life of less than 1 h.[1,20,24]

Under normal translation initiation, the small ribosomal subunit associates with the ternary complex (eIF2, GTP, and Met-$tRNA_i^{Met}$), binds to mRNAs, and scans for open reading frames (ORFs). Once an ORF is found, the ternary complex dissociates for the ribosome to form and initiate translation. The small ribosomal subunit must bind to a new ternary complex each time to re-initiate translation and build ribosomes. When the ISR is activated, eIF2B (part of the ternary complex) becomes inhibited and reduces available ternary complexes, inhibiting global protein synthesis. ATF4 uses transcript-level regulation to evade this inhibition by using three upstream ORFs (uORFs).[25–27] uORFs are nucleotide sequences, upstream or overlapping with the coding sequence (CDS) of a gene, that can affect its translation efficacy.[28] In *ATF4*, uORF1 is 6 nt long, and uORF2 is 12 nt long. uORF1 and 2 are located within the 5′ UTR and do not overlap the CDS. In contrast, uORF3 is 180 nt long and overlaps with the *ATF4* CDS. In normal, unstressed

[1]Department of Human Genetics, University of Utah School of Medicine, Salt Lake City, UT, USA; [2]Department of Molecular Biosciences, University of Kansas, Lawrence, KS, USA
[3]All editorial responsibility for this paper was handled by an associate editor of the journal
[4]Lead contact
*Correspondence: hansmdalton@ku.edu (H.M.D.), cchow@genetics.utah.edu (C.Y.C.)
https://doi.org/10.1016/j.xhgg.2026.100666.
© 2026 The Author(s). Published by Elsevier Inc. on behalf of American Society of Human Genetics.
This is an open access article under the CC BY license (http://creativecommons.org/licenses/by/4.0/).

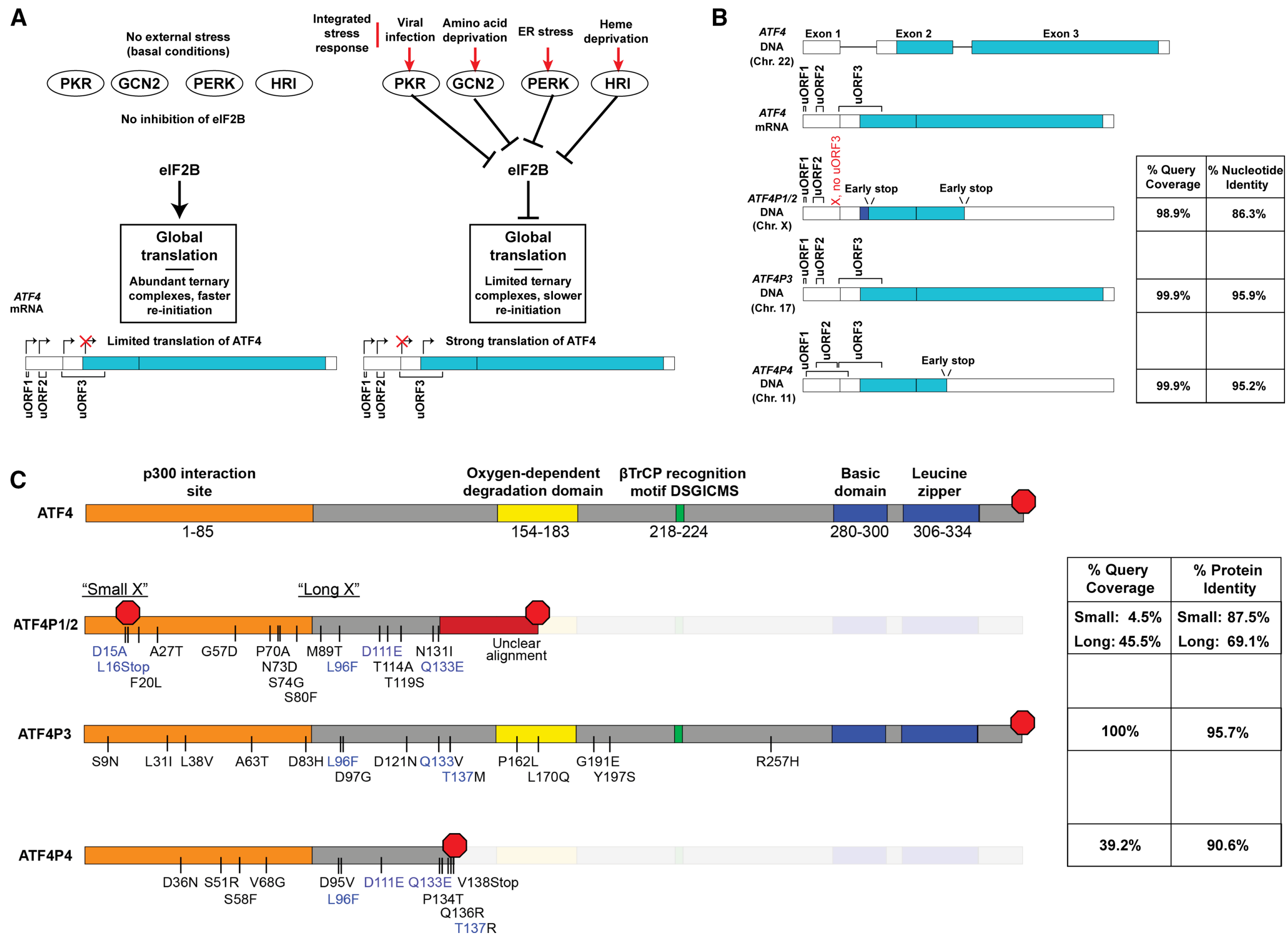

Figure 1. Characterizing four duplications of *ATF4*

(A) A simplified schematic of the integrated stress response (ISR) and translation of ATF4. Under normal conditions, the ISR is not activated, and global cell translation is normal. Due to the overlapping uORF3, ATF4 translation is limited due to abundant ternary complexes skipping the *ATF4* CDS. Under stress conditions, the ISR is activated, thereby inhibiting eIF2B and global cell translation. The limited ternary complexes delay re-initiation until after the uORF3 start site, thereby preferentially translating the *ATF4* CDS instead. Teal sections indicate the CDS of *ATF4*.

(B) Cartoons of genomic *ATF4*, *ATF4* mRNA, and genomic *ATF4P1–P4*. We list the current status of uORFs here, including when it is lost in *ATF4P1/P2*. We also list the % coverage and identity of each duplicate nucleotide sequence (minus flanking TSDs) when compared to *ATF4*. Teal sections indicate the CDS of ATF4, and the blue band is a short 16-aa-encoding segment in *ATF4P1/2* (Small X).

(C) A diagram of each translated retrocopy compared to the translated Ensembl Canonical transcript of *ATF4*. Amino acid changes that occur in more than one retrocopy are highlighted in blue. We also list the % coverage and identity of each translated protein sequence when compared to translation of *ATF4* mRNA (cDNA).

conditions, ribosomes first form on uORFs 1 or 2 and complete this short translation. Because ternary complexes are abundant under non-stressed conditions, the small ribosomal subunit continues scanning, and it usually finds and translates uORF3. Because uORF3 overlaps with the CDS, this process often "skips" over *ATF4* and, consequently, ATF4 protein is rarely produced.[25–27] However, under the ISR, there are not enough ternary complexes to quickly re-initiate translation at uORF3, so scanning instead continues to the CDS ORF, at which point enough time has passed to allow a new ternary complex to bind. Thus, under the ISR, full-length ATF4 is translated more frequently.[25–27] These uORFs are highly conserved in vertebrates, indicating their importance in ATF4 regulation.[26]

ATF4 has four gene duplications in humans (annotated as pseudogenes *ATF4P1–P4*) that have not been characterized. While rare, gene duplications can maintain or even gain functionality.[29–31] One class is retroduplications (retrocopies), which are reverse transcribed (via transposon activity[32,33]) from the mRNA of "parent" genes and inserted back into the genome. When retrocopies are found to be functional, they are labeled as retrogenes,[34] such as HAPSTR2[35] (MIM: 301145),[3] retroCHMP3,[36] PTENP1[37,38] (MIM: 613531),[3] and PGK2[39–41] (MIM: 172270).[3] To be considered a functional retrogene, these previous studies have shown the gene is transcribed and causes a biological outcome through its mRNA or protein. This could be with or without interaction with its original parent gene. If a gene duplication is functional and beneficial, evolution

can maintain it in the genome.[29] One major source of evolutionary pressure on the ISR comes from viruses.[42,43] For example, hepatitis B hijacks ISR signaling proteins in order to prevent downstream apoptosis.[44] Adenovirus produces decoy double-stranded RNAs (dsRNAs) that sequester the ISR kinase PKR without activating it, thereby inhibiting its anti-viral activity.[42,45] These pressures have led to evolutionary selection in ISR machinery, such as in PKR, where it has evolved to evade viral mimicry.[46] Given that ATF4 is the critical downstream transcription factor of the ISR, such evolutionary pressure could select for these *ATF4* duplications if they influence ISR function.

Here, we demonstrate that the four *ATF4* duplications are retrogenes. These retrogenes span evolutionary events across at least 37 million years in vertebrates. There is evidence of divergent and convergent evolution, with one *ATF4* retrogene truncation arising independently in at least three different primate species. Human retrogenes are expressed under normal conditions and ISR activation, are degraded by the same machinery as *ATF4*, and can alter the expression of ATF4 target genes. Given their evolutionary history and function in cells, these retrogenes are likely important in the highly conserved ISR pathway in vertebrates.

Materials and methods

Databases accessed for gene queries, expression, and protein folding

We used the University of California Santa Cruz (UCSC) genome browser to identify retrocopies of *ATF4* by using BLAT[47] to search using the species-specific endogenous parental *ATF4* gene. We identified retrocopies, with a BLAST-like Alignment Tool (BLAT) matching score of at least 100, by the presence of a poly(A) tail and/or by a lack of introns. We noted cases of uncertainty, such as with short contigs or poor (e.g., "N"-rich) sequencing around potential retrocopies. In species where *ATF4* was not annotated, we used a closely related species (when possible) to predict the parental *ATF4* location in the queried species then used that section to search that species for potential retrocopies. We also noted any duplications of *ATF4* in Table S2, but only for annotation purposes, and we do not discuss them here.

We annotated exons and introns of human *ATF4* by referencing the Ensembl genome database.[48] We specifically used the canonical *ATF4* transcript ENST00000674920.3 (ATF4-206). We referenced the UniProt database to confirm post-translational modification sites.[49] We used MacVector (v.18.2.5.) to help annotate and visualize DNA sequences.

We predicted protein structures using AlphaFold 3 using default settings.[50] We downloaded .CIF files of each protein and oriented them using PyMOL (The PyMOL Molecular Graphics System, Version 2.2.6, Schrödinger). To help visualize their structures, we used PyMOL to color specific amino acids for Figure S1.

To create our species tree, we first generated our .tre file using the "rotl" package in RStudio (version 2023.12.1+402, RStudio Team (2020). RStudio: Integrated Development for R. RStudio, Boston, MA; http://www.rstudio.com/). We uploaded this file to the interactive tree of life[51] to correct minor labeling errors and to sort species.

Cells

RPE-1 cells (hTERT RPE-1, CRL-4000, American Type Culture Collection [ATCC]) were a gift from Drs. Michael Boland and David Goldstein (Columbia University). HEK293T cells were gift from Dr. Charles Murtaugh (University of Utah).

We maintained RPE-1 cells in DMEM/F-12 media (Gibco, Thermo Fisher catalog [cat.] 11320033) supplemented with 10% fetal bovine serum (FBS) (Gibco, Thermo Fisher cat. 26140079) and 0.01 mg/mL hygromycin B (Sigma-Aldrich, cat. H3274, diluted in water). We maintained HEK293T and HeLa cells in DMEM with pyruvate (Gibco, Thermo Fisher cat. 11995065) supplemented with 10% FBS (Gibco, Thermo Fisher cat. 26140079). We grew both cells in cell incubators set to 37°C and 5% CO_2.

Determining ATF4 retrocopy expression via database and qPCR

We used the Genotype-Tissue Expression (GTEx) Project to determine expression levels of *ATF4* retrocopies (*ATF4P1–P4* are annotated on GTEx). We queried the Oxford Nanopore Technologies long-read data (GENCODEv26-aligned) for annotated transcripts of *ATF4P1* (ENST00000443279), *ATF4P2* (ENST00000601944), *ATF4P3* (ENST00000433309), *ATF4P4* (ENST00000438498), *PTENP1* (ENST00000447117), *PGK2* (ENST00000304801, ENST00001129597), and *HAPSTR2* (ENST00000453380). GTEx was supported by the Common Fund of the Office of the Director of the National Institutes of Health and by NCI, NHGRI, NHLBI, NIDA, NIMH, and NINDS. The data used for our analyses here were obtained from the GTEx Portal on under the dbGaP accession phs000424.v10.p2 on 02/17/2025 and the long-read data on 06/20/2026. We used the UCSC genome browser[47] to access tracks containing ENCODE data for chromatin marker and candidate *cis*-regulatory element (cCRE) data,[52] transcription start site markers using CAGE data,[53] and JASPAR motifs[54] of common ISR and unfolded protein response (UPR) transcription factors (ATF4, ATF3, CEBPA, CEBPA/DDIT3, CEBPB, XBP1, and ATF6).

To design primers for *ATF4*/retrocopy expression, we first aligned *ATF4* and its retrocopies using ClustalOmega.[55] As we needed to use primers specific to each copy, without binding each other, we focused on DNA segments with low similarity between each retrocopy. When such areas were small, we used that low similarity area as the 3′ end of each primer, as this is the most important for primer binding/synthesis[56] (see Table 1). We used qPCR primers for *ATF4* target genes from PrimerBank[57] or designed them using Primer3Plus.[58] Primers were ordered from IDT DNA (Coralville, IA).

To generate RNA, we first grew cells in six-well plates until ~80% confluent. We then added TRIzol reagent (15596026, Invitrogen) to wells and scraped cell lysates into microcentrifuge tubes. We vortexed tubes for at least 15 s, and we stored them at −80°C for at least 24 h before further processing. We used the DirectZol kit (Zymo Research, cat. R2052) to extract RNA and the ProtoScript II First Strand cDNA Synthesis Kit (NEB, cat. E6560L) to convert RNA to cDNA. We analyzed gene expression using ATF4/retrocopy-specific primers (Table 1), PowerUp SYBR Green Master Mix (Fisher Scientific, cat. A25918), and QuantStudio 3 for analysis (Thermo Fisher).

dN/dS and ORF decay modeling

To determine the non-synonymous to synonymous (dN/dS) ratios of retrocopies, we used the KaKs_Calculator 2.0.[59] We used ORFs of human *ATF4P1–P4* compared to either human *ATF4* or

Table 1. List of primers used

Name	Nucleotide sequence used	Purpose
**Primers for Gateway cloning**		
ATF4 forward	CTCTCTCCTTTCGTGAGGCC	for amplifying region around *ATF4* for gel extraction
ATF4 reverse	TGTTCAGGTAACAAGTGAAACCT	
ATF4P3 forward	CCGGGCCACTACCTCTTACT	for amplifying region around *ATF4P3* for gel extraction
ATF4P3 reverse	GGTGCAATGGCTCACAACTG	
ATF4P4 forward	CAAGATCCCCGATGAGGAGC	for amplifying region around *ATF4P4* for gel extraction
ATF4P4 reverse	CTCAACTTCCTGGGCTCGAG	
ATF4P1/2 forward	CCCCCAAGTCTTAACCCATT	for amplifying region around *ATF4P1/2* for gel extraction
ATF4P1/2 reverse	AGCGTAGTGCAGAAGGGAAA	
ATF4/*ATF4P3/ATF4P4* forward	CACCGCCACCATGACCGAAATGAGC	for creating the Gateway cloning insert and fixing Kozak
ATF4/*ATF4P3* reverse	GGGGACCCTTTTCTTCC	
ATF4P4 reverse	CCGTCTGGGGGTCTCCT	
ATF4P1/2, Long X forward	CACCGCCACCATGTCCCCCCTTGA	
ATF4P1/2, Long X reverse	AAGGAATAATCTGGAGTG	
ATF4P1/2, Small X	CACCGCCACCATGACCGAGATGAG CTTCCTGAGCAGCGAGGTGTTGGTG GGGGCT	synthesized directly (IDT) for use in Gateway cloning
**Primers to differentiate ATF4 and retrocopy transcripts**		
ATF4 forward	TGACCACGTTGGATGACACT	qPCR
ATF4 reverse	GGGAAAGGGGAAGAGGTTGT	qPCR
ATF4P3 forward	CCCTAGTCCAGGAGACTAATAAGGT	qPCR
ATF4P3 reverse	AGGGATCATGGCAACGTAAG	qPCR
ATF4P4 forward	CTGGTGAGTGCAAAGAGCTG	qPCR
ATF4P4 reverse	ACACAGCTACAGCAGAGG	qPCR
ATF4P1/2 forward	GGCTCTCCCTCTACCTCCAG	qPCR
ATF4P1/2 reverse	GCTGTTTGGTTTTGCTCCAT	qPCR
***ATF4* target genes**		
ATF3 forward	CCTCTGCGCTGGAATCAGTC	qPCR PrimerBank ID: 346223459c1
ATF3 reverse	TTCTTTCTCGTCGCCTCTTTTT	
TRIB3 forward	AAGCGGTTGGAGTTGGATGAC	qPCR PrimerBank ID: 41327717c1
TRIB3 reverse	CACGATCTGGAGCAGTAGGTG	
XPOT forward	CAAGTCTTCGCCTTGCTTTTTG	qPCR PrimerBank ID: 317108146c2
XPOT reverse	CCCTTGGATTTAGGTCCACTACT	
SARS1 forward	CAGCCCTCATCCGAGAGAC	qPCR PrimerBank ID: 300795157c1
SARS1 reverse	TCTGCCCGAAATCTACATCGT	
ERO1A forward	GGCTGGGGATTCTTGTTTGG	qPCR PrimerBank ID: 7657068c1
ERO1A reverse	AGTAACCACTAACCTGGCAGA	
GAPDH forward	ACAACTTTGGTATCGTGGAAGG	qPCR PrimerBank ID: 378404907c2
GAPDH reverse	GCCATCACGCCACAGTTTC	

our reconstructed ancestral versions of *ATF4*, and we set the parameters to use the Yang and Nielson (YN) methodology.[60]

We aligned *ATF4* sequences using Multiple Sequence Alignment by Log-Expectation (MUSCLE 3.8)[61] of either apes or Catarrhini to represent ancestral copies of species 14.8 million years ago (mya) and 37.5 mya, respectively. We then created estimates of ancestral *ATF4* by inputting these MUSCLE-aligned sequences into EMBOSS Cons[61] to create

Table 2. Kozak sequences in ATF4 and retrocopies

Gene	Endogenous Kozak	Modified Kozak
ATF4	CGCAACATGACC	GCCACCATGACC
ATF4P3	CGCAACATGACC	GCCACCATGACC
ATF4P4	CGCAACATGACC	GCCACCATGACC
ATF4P1/2, Small X	TGCACCATGACC	GCCACCATGACC
ATF4P1/2, Long X	GGCTTGATGTCC	GCCACCATGTCC

Native *ATF4*/retrocopy Kozak sequences required modification to induce correct protein expression in our cells.

consensus sequences using minimal manual annotation (see Table S3).

We used the Python script "mutator" to simulate the decay of *ATF4* retrocopies under neutral evolution, and "orf_scanner" to count ORFs.[62,63] We modified the code of the mutator script to use a Poisson distribution (rather than Gaussian) to better model the relatively short evolutionary times of these retrocopies (see new code at https://github.com/hansmdalton/pedestal_fork). As we are most focused on the human retrocopies, we used human substitution, insertion, and deletion rates as determined from the literature[63,64]: substitution = 1.16e−8, insertion = 2e−10, deletion = 5.5e−10. We used a generation time of 25 years for *ATF4P3* and *ATF4P4* to represent apes,[65] and we used a generation time of 10 years for *ATF4P1/P2* to represent monkeys within Catarrhini that still maintain this retrocopy.[66–68] We derived the estimates of 14.8 mya divergence of orangutans and 37.5 mya divergence of *Platyrrhini* from the literature.[69–73] We simulated ORF decay in 10,000 replicates every 50,000 years for 50 million years.

Starting from our estimated ancestral copy (Table S3) at 14.8 million years (my) or 37.5 my, we determined probabilities of each lineage-specific branch terminus retaining the intact retrocopy ORF being tested (e.g., full 351 amino acids [aa] for ATF4P3, or >130 aa for ATF4P1/2 Long X). To prevent double-counting branches, we sectioned the phylogenetic branches into non-overlapping segments for testing species probabilities. To determine the overall probability, we then multiplied these probabilities together for each ORF. For amino acid changes in the DNA-binding domain of *ATF4P3*, we used Biopython[74] to translate each permutation to look for any non-synonymous changes or indels. To look for any nucleotide changes in *ATF4P2*, we ran the same mutator script as above, but then we counted (using Python) the presence of any nucleotide change or indels. We then plotted all of these results using Rstudio[75] and annotated graphs in Adobe Illustrator.

Target-site duplication identification

The presence of poly(A) sequences and the lack of introns within the sequences of retrocopies indicates that they likely arose from retrotransposons.[34,76] This is typically driven by long interspersed element-1 LINE-1) elements, which leave a duplicated sequence artifact flanking each retrocopy.[34,76] We manually assessed the ~100 bp downstream of the poly(A) sites following the 3′ UTR (as determined from parental *ATF4*). We used this sequence to identify identical sequences upstream of the 5′ UTR. When we identified a likely target site duplication (TSD) site, we checked this TSD against the rest of the species for this same sequence, refining the sequence as needed.

ISR stress treatments

We grew wild-type (WT) RPE-1 cells to ~80% confluency for the experimental treatment day. We treated cells with either 0.1 μM thapsigargin (Tg) (Sigma cat. S7400) for 8 h (vehicle = DMSO) or 10 μM sodium arsenite (As) (Sigma cat. T9033) for 6 h (vehicle = water). We plated 3–5 biological replicates of WT RPE-1 cells in six-well plates (As) or 10-cm plates (Tg). After treatment, we collected cells in TRIzol and performed qPCR analysis as above.

Lentivirus production and transfection

As *ATF4* retrocopies have high similarity to each other at the nucleotide level, we employed two sequential PCR reactions to specifically amplify each retrocopy for cloning (Table 1). Due to differences in introns, the cloned sequence for *ATF4* was derived from RNA (cDNA), while the retrocopies were derived from genomic DNA. We derived genomic DNA from HeLa cells and RNA from human fibroblasts[77] (gifts of Dr. Nels Elde, University of Utah). We converted RNA to cDNA using the ProtoScript II First Strand cDNA Synthesis Kit (NEB, cat. E6560).

First, we used primers ~100–500 bp up- and downstream of each *ATF4* retrocopy to amplify that region. We then gel extracted (QIAquick Gel Extraction Kit, cat. 28704) this product to purify it from other genomic DNA. We used this as the product for further Gateway cloning using primers specific to each gene. Because the Small X insert was so short (55 bp), we instead ordered this entire sequence from IDT DNA (Coralville, IA) ready for further Gateway cloning. We sequenced all inserts and final products to ensure these were correct (Sanger sequencing via the University of Utah HSC DNA Sequencing Core).

For all copies of *ATF4* (including the parental gene *ATF4* itself), we observed no protein expression when we directly cloned either the CDS alone or the 5′ UTR plus CDS (performed in both RPE-1 and HeLa cells). To help resolve this, we designed primers to modify the native Kozak sequence[78] upstream of the first codon while maintaining the coding sequence integrity. Using these modified Kozak sequences (Table 2), we observed protein expression of all clones.

We used the Gateway cloning system (Invitrogen, Thermo Fisher cat. K240020) to generate the entry vectors used for creating ATF4 overexpression (OE) cell lines. We then used LR clonase (Invitrogen, Thermo Fisher cat. 11791020) to transfer our clones into a lentiviral plasmid containing a cytomegalovirus (CMV) promoter to drive the OE and a C-terminal V5 tag (Addgene, pLenti6.2-ccdB-3xFLAG-V5, Plasmid #87071). We used a C-terminal tag to avoid future conflicts with 5′ UTR/Kozak/uORF analyses. We then generated lentivirus particles in HEK293T cells via FuGENE 6 Transfection Reagent (Promega cat. E2693) using psPAX2 and pCAG-VSVG packaging plasmids (Addgene plasmid #12259, and Addgene #35616, respectively; gifts of Dr. Charles Murtaugh, University of Utah).

We incubated RPE-1 cells with our generated lentivirus particles along with 8-μg/mL polybrene (gift from Dr. Charles Murtaugh, University of Utah). After 48 h, we selected for transfected polyclonal cell populations using 10-μg/mL blasticidin (Gibco, Thermo Fisher cat. R21001). We then selected for monoclonal populations by picking cell colonies to individual plates where we continued blasticidin selection and ensured single-colony expansion. We then performed western blot analyses (see below) to check for accurate and robust protein production of each cloned retrocopy.

Proteasome inhibition

To induce proteasome inhibition, we first grew RPE-1 cells to 80%–90% confluency. We then replaced their regular media with media containing either 0 or 100 nM bortezomib (Btz) (both with 0.1% DMSO). After 6 h of exposure to Btz, we collected cell protein for western blot or cells in TRIzol for RNA extraction and downstream qPCR analysis.

Western blots

To collect protein, we washed cells with PBS, then we treated them with RIPA buffer supplemented with 1× Protease Inhibitor Cocktail (Cell Signaling Technologies, cat. #5871). We lysed cells for 30 min at 4°C under agitation, then centrifuged them at 14,000 × *g* for 20 min. We extracted supernatants and determined protein concentration using the bicinchoninic acid (BCA) assay (Pierce BCA Protein Assay Kit, cat. 23227). We then froze samples at −80°C for later use.

To denature proteins, we added 6× loading buffer supplemented with β-mercaptoethanol (Sigma, cat. M6250) and heated them at 95°C for 5 min. We resolved proteins using a 12% Criterion XT Bis-Tris Protein Gel (Bio-Rad #3450118) and XT MES Running Buffer (Bio-Rad, cat. #1610789). We detected proteins using anti-V5 (Cell Signaling Technologies, cat. 13202, 1:1,000), anti-ATF4 (Proteintech, cat. 10835-1-AP, 1:800), anti-beta-actin (Santa Cruz Biotechnology, cat. sc-47778, 1:200) primary antibodies, and anti-rabbit and anti-mouse secondaries ("DyLight," Cell Signaling Technology cat. 5151 and 5470, respectively, 1:10,000). We used the Precision Plus Protein All Blue Standards for our ladder (Bio-Rad, cat. #1610373). We visualized blots using an Odyssey DLx imager (LI-COR).

Statistics and graphs

We performed statistics and created figures using GraphPad Prism (version 10 for Windows, GraphPad Software, Boston, MA, USA; www.graphpad.com). We also utilized RStudio (version 2023.12.1+402, RStudio Team (2020). RStudio: Integrated Development for R. RStudio, Boston, MA; URL http://www.rstudio.com/).

Results

Humans have four retrocopies of *ATF4*

There are four annotated *ATF4* duplications: *ATF4P1*, *ATF4P2*, *ATF4P3*, and *ATF4P4*. All of these duplications lack introns and end in truncated poly(A) tails—key characteristics of retrocopies[34,79] (Figure 1B; Table S1). Retrocopies often originate from LINE-1 retrotransposons in mammals,[32,33] and these leave short TSDs flanking each gene.[33,80] We examined the region around each retrocopy and found evidence for TSDs in all of them (Table S1). Thus, *ATF4P1–4* are retrocopies that emerged through LINE-1 retrotransposons.

We compared the nucleotide sequence and putative protein translation of each retrocopy to its parent gene, *ATF4* (Figures 1B and 1C, AlphaFold predicted structures in Figure S1).

ATF4P2 and ATF4P1

ATF4P2 and *ATF4P1* are located on the X chromosome. Here, there is a larger ape-specific chromosomal reverse tandem duplication event.[81] The proximal border of this duplication is in the middle of the gene, *IKBKG* (MIM: 300248),[3] resulting in the distal end containing a truncated duplication (the pseudogene, *IKBKGP1*). As *ATF4P1* is closer to the more recent *IKBKGP1*, it must be duplicated from *ATF4P2*, which is closer to *IKBKG*. This duplicated region is highly invariant. As such, human *ATF4P2* and *ATF4P1* are 100% identical at the nucleotide level from the first uORF to the poly(A) tail (Table S1), and we refer to them together as *ATF4P1/2* throughout. While *ATF4P1* was not directly derived from a retroduplication event, it nevertheless was duplicated from the retrocopy *ATF4P2*, so we refer to it as a retrocopy/retrogene throughout.

ATF4P1/2 maintain uORF1 and uORF2, but they have lost uORF3 due to a new stop codon (Figure 1B). This very early premature stop codon would result in a putative 15-aa product. However, *ATF4* and *ATF4P1/2* have a secondary in-frame start site after 48 bp. This is immediately following the premature stop in *ATF4P1/2* and encodes a 170-aa product ending with multiple insertions that result in amino acid changes and a premature stop codon after the p300 interaction domain (Figures 1C and S1). Going forward, we will refer to these products as Small X (15 aa) and Long X (170 aa), respectively. At 4.5% query coverage (15 aa), ATF4P1/2 Small X has 87.5% identity with ATF4. At 45.5% query coverage (170 aa), ATF4P1/2 Long X has 69.1% protein identity with ATF4 (Figure 1C).

ATF4P3

ATF4P3 is located on chromosome (Chr.) 17 in an intron of the *RNF157* gene (Figure S2) (MIM: 621044).[3] Because *ATF4P3* was inserted in the same strand orientation as *RNF157*, it might utilize the *RNF157* promoter or its regulatory sites. At the nucleotide level, all of its upstream ORFs (uORFs) are intact (Figure 1B), suggesting similar translational regulation to *ATF4*. *ATF4P3* encodes for a putative full-length ATF4 protein with zero nonsense changes. At 100% query coverage, ATF4P3 has 95.7% protein identity with ATF4 (Figure 1C). Notably, the majority of its missense changes (12/15) occur in the first half of the gene, with fewer in the second half (3/15), and none are present in the basic leucine zipper domains (Figure 1C). Because the ATF4 leucine zipper domain contains a nuclear localization signal (KKLKK[82]), it is likely that ATF4P3 retains its nuclear localization and DNA-binding ability. Overall, *ATF4P3* is nearly completely intact (∼95%) at both the nucleotide (Figure 1B) and protein level (Figures 1C and S1).

ATF4P4

ATF4P4 is located on Chr. 11. Its uORFs 1 and 2 are extended due to changes in their stop codons (Figure 1B). uORF1 now overlaps both uORF2 and uORF3. This uORF1 overlap likely results in inhibiting normal ribosomal binding to uORF3, which usually causes the CDS to be skipped,[25–27] and this likely would release some uORF inhibition of the *ATF4P4* CDS. ATF4P4 contains a premature stop codon that occurs after the p300 interaction domain, and this results in a 138-aa product. Despite

this truncation, *ATF4P4* is nearly completely intact at the nucleotide level (~95% identity). While ATF4P4 lacks both degradation domains and its DNA-binding domain at the protein level, its p300 interaction domain remains intact (Figures 1C and S1). This suggests that ATF4P4 would lack targeted degradation while still maintaining its ability to bind p300. Within the 138-aa product (39.2% query coverage), ATF4P4 has 90.6% amino acid identity with ATF4 (Figure 1C).

Several sites of post-translational modification in ATF4 are changed in these retrocopies. ATF4P1/2 Long X has differences in two phosphorylation sites (T114A, T119S). These are two of four phosphorylated threonine residues, along with T107 and T115, important for protein stability.[83] Mutation of all four of these threonine residues causes a strong increase in ATF4 stability. It is possible that the T119S site is still phosphorylated (as it is a serine). However, the T114A change should remove the phosphorylation site, thereby increasing the stability of ATF4P1/2 Long X protein. ATF4P3 has a change in a proline residue in the ODD domain (P162L), one of five prolines important for proper ODD domain-induced degradation.[20] This may lead to impaired degradation of ATF4P3 under normoxia, as this is observed for the same mutation in ATF4.[20] ATF4P4 has a change in a phosphorylation site (S58F), and mutations in this same site in ATF4 lead to reduced transcription factor activity.[84] Given that ATF4P4 is missing its DNA-binding domain, it is unclear what effect this amino acid difference has on ATF4P4. Overall, *ATF4P1–4* are relatively intact and have high nucleotide and protein identity with *ATF4*.

Conserved and independent retrocopies of *ATF4* are common in mammals

If *ATF4* retrocopies are well-conserved across many species, it suggests they are under non-neutral selection. As such, we examined the evolutionary history of each *ATF4* retrocopy. We analyzed *ATF4* retrocopies through manual BLAT queries of 97 species, from yeast to humans, derived from UCSC-curated genome assemblies (Table S2; UCSC genome browser[47]).

ATF4P2 and ATF4P1

Retrotransposition of *ATF4P2* occurred in a common ancestor of apes and Cercopithecidae, the monkeys of Africa and Asia, approximately 37.5 mya (Figures 2A and S3; Table S2). In the six Cercopithecidae species we examined, *ATF4P2* contains the same ancestral early stop for its Long X protein, which results in a putative 131-aa product (Figure 2A). This is different from what is observed in apes, where they range from 85 to 170 aa in length. The only intact protein domain in *ATF4P2* Long X is the p300-binding domain.

ATF4P1 is an ape-specific duplication of the *ATF4P2* retrocopy. It likely arose ~14.8 mya because it is present in humans, chimps, bonobos, and gorillas but not in orangutans or gibbons[69–73](Figure 2B). Human and gorilla *ATF4P1* genes have a putative Long X protein of 170 and 160 aa, respectively. In chimps and bonobos, their *ATF4P1* Long X sequence is truncated with an early stop resulting in a putative 14-aa-long product (different from Small X protein). Bonobos also have an additional retrocopy duplication in tandem with its *ATF4P2* and *ATF4P1* (Figures 2B and S3; Table S1), resulting in three total copies. This additional bonobo duplication has 98% nucleotide identity with *ATF4P2*, and only 91% with *ATF4P1*, so it most likely also arose from *ATF4P2* sometime in the last ~2 my.

Humans, chimps, and bonobos have a 30- to 34-bp internal duplication in *ATF4P2* and *ATF4P1* between the p300 interaction and ODD domains (Table S1). In humans, this is exactly 30 bp (and thereby in-frame) in *ATF4P2* and *ATF4P1*, causing its Long X protein to be 10 aa longer. However, this segment is 32 bp long in chimp *ATF4P2* and results in an early stop. The duplication likely has no impact on bonobo *ATF4P2* or *ATF4P1* or its additional duplication, or chimp *ATF4P1*, as other indels cause earlier stop codons. Unexpectedly, this ~30-bp duplication is not present in gorilla *ATF4P2* or *ATF4P1* (2019 and 2016 genome assemblies via UCSC genome browser). As it is not present in orangutans or Cercopithecidae either, it is unlikely due to a deletion event in gorillas, and it is instead likely a new duplication arising after the gorilla split. However, because it is present in *ATF4P2* and *ATF4P1* (and the additional duplication in bonobos), this suggests the unlikely occurrence of two independent duplication events of this segment in *ATF4P2* and *ATF4P1* in the common ancestor of humans, chimps, and bonobos.

Given the divergence time of 14.8 my, and a human mutation rate of $\sim 1.1 \times 10^{-8}$/generation,[85] we expected to find ~9 nucleotide changes between *ATF4P2* and *ATF4P1*. However, human *ATF4P2* is identical to *ATF4P1* at the nucleotide level. As described above, this region of the genome is highly invariant after a larger genomic duplication event.[81] One possibility is that selection on something in this region is maintaining identity between the duplicates. Alternatively, given the tandem duplication and 100% identity, this could be due to interlocus gene conversion, where one DNA sequence is replaced by a nearby paralog.[86] However, compared to other apes examined, human *ATF4P2* and *ATF4P1* are the only unchanged pair. Chimps, bonobos, and gorillas all have nucleotide differences between *ATF4P2* and *ATF4P1*, and the third copy in bonobos is also different from its *ATF4P2* and *ATF4P1* (Figures 2A and 2B; Table S1). Thus, such interlocus gene conversion would likely be specific to humans.

Of note, chimps and bonobos are very closely related species,[87] yet their *ATF4P2* and *ATF4P1* retrocopies contain several nucleotide and protein differences relative to each other. For example, while both have a putative truncated 14-aa-long product in *ATF4P1*, these were independent, convergent events (22Gdel in bonobos and 5Cdel in chimps). In addition, ATF4P2 is truncated at a

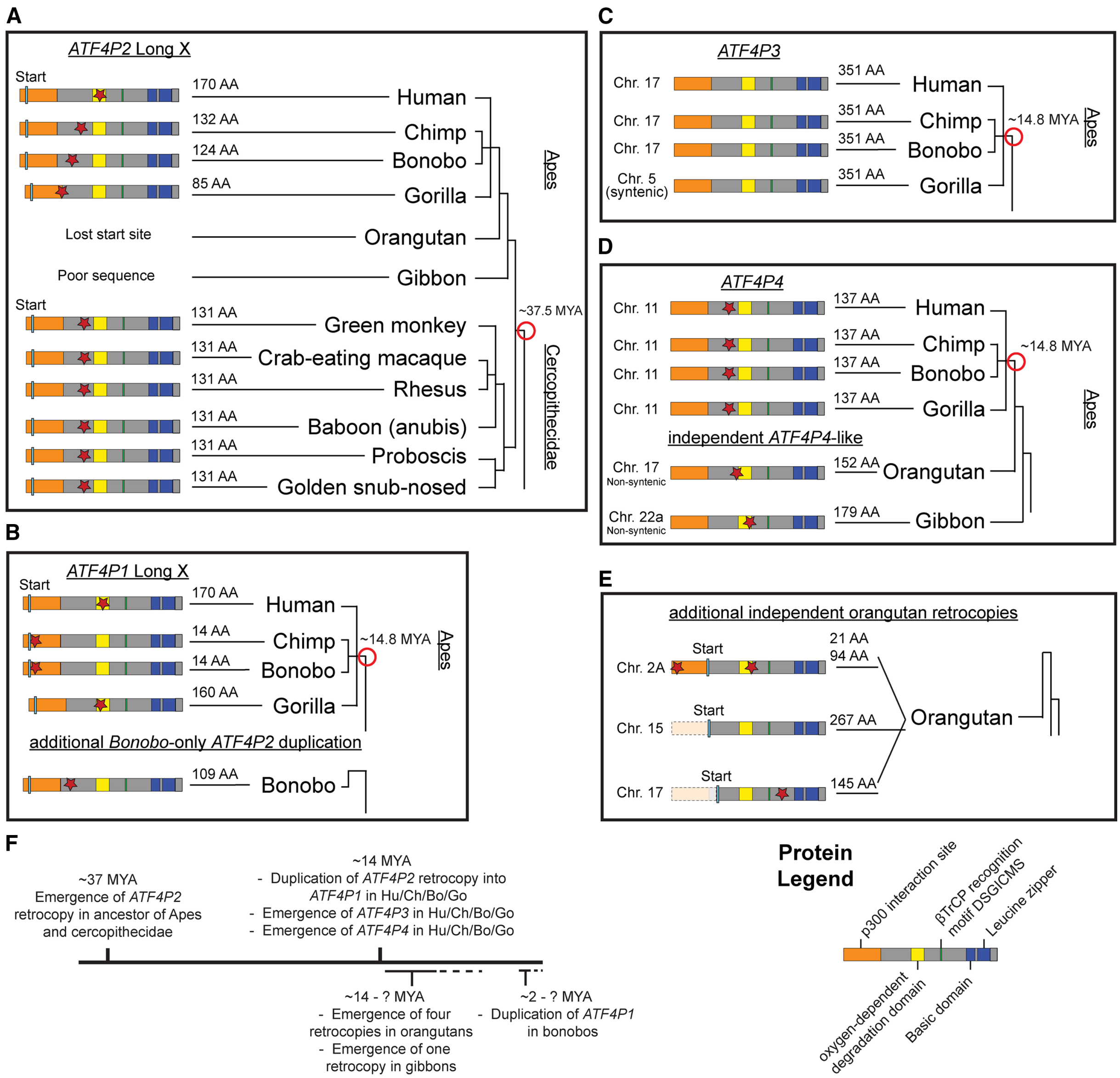

Figure 2. Catarrhini species tree comparisons of *ATF4* retrocopy and duplicate retrocopy proteins

(A) *ATF4P2* arose ~37.5 mya in a common ancestor of Catarrhini (apes and Cercopithecidae, the monkeys of Africa and Asia). There is a short Small X peptide (ending at the blue bar) followed by a longer ORF (Long X) that resembles the truncated *ATF4P4*. Note that orangutan only lost the start site of *ATF4P2* Long X, but they still maintain the highly truncated *ATF4P2* Small X (Table S1). Cercopithecidae all contain the same truncating stop site resulting in a 131-aa product.
(B) *ATF4P1* resulted from a duplication of *ATF4P2* ~14.8 mya. In humans, it is an exact copy even at the nucleotide level, while chimps, bonobos, and gorillas have various levels of truncation. Chimps and bonobos have a highly truncated Long X in *ATF4P1*, but they maintain it in *ATF4P2* (see A). Bonobos also have an additional duplication of *ATF4P2* with its own unique truncation.
(C) *ATF4P3* arose in a common ancestor of apes ~14.8 mya, and it is fully intact at the amino acid level in each species we examined.
(D) *ATF4P4* arose in a common ancestor of apes ~14.8 mya, and it carries an early stop that truncates the protein to 137 aa. There are two independent *ATF4* retrocopies in orangutans and gibbons with similar truncations to *ATF4P4* but are non-syntenic.
(E) Orangutans have three independent (non-syntenic) retrocopies of *ATF4*. Two are genomically truncated (Chrs. 15 and 17), and Chr. 2A carries two early stop codons. This Chr.17 retrocopy is likely a partial duplication of the ATF4P4-like orangutan retrocopy in (D) given its proximity.
(F) Timeline of approximate emergence of new retrocopies and their duplications. AA, amino acid; mya, million years ago.

different amino acid length due to various nucleotide differences between chimps and bonobos (Figure 2A; Tables S1 and S2).

ATF4P3

ATF4P3 arose in a common ancestor of humans, chimps, bonobos, and gorillas (Figures 2C and S3; Tables S1 and S2). In each species, *ATF4P3* has a full protein-length ORF, although each species has differences in amino acid changes (Figure S4). Similar to humans, there are no changes to the DNA-binding domain of ATF4P3 in chimps compared to ATF4 (Figure S4). Bonobo ATF4P3 has one change (K315Q) in its DNA-binding domain, while gorilla

ATF4P3 has two changes (R296C, N317S). We queried how these amino acid changes might impact protein function by using PolyPhen[88] and SIFT.[89] K315Q in bonobo is predicted to be "benign" and "tolerated" by PolyPhen and SIFT, respectively. For the gorilla changes, both are predicted as "probably damaging" and "affecting protein function" by PolyPhen and SIFT, respectively. Given its sequence is identical in humans and chimps, and that the bonobo change is predicted as benign/tolerated, this domain is likely functional if it is translated.

ATF4P4

Similar to *ATF4P3*, *ATF4P4* arose in a common ancestor of humans, chimps, bonobos, and gorillas (Figures 2D and S3; Tables S1 and S2). In all species, ATF4P4 retrocopies are truncated by the same early stop codon, resulting in a 137-aa product. We also found two similarly truncated (but independent/non-syntenic) "*ATF4P4*-like" retrocopies in both orangutans and gibbons, creating 152- and 179-aa products, respectively (Figure 2D). Thus, this truncated variant of an ATF4 retrocopy has arisen at least three independent times. Similar to ATF4P1/2, these truncated retrocopies only contain an intact p300-binding domain. Taken together, humans, bonobos, and gorillas all have three intact ATF4 retrocopies containing only the p300-binding domain (chimps have two). If expressed, this suggests some benefit to having extra copies of a p300-binding peptide.

Independent orangutan ATF4 retrocopies

Orangutans also contain three additional *ATF4* retrocopies that are non-syntenic to any other retrocopy we observed (Figures 2E and S3; Tables S1 and S2). These retrocopies are unlike *ATF4P1–P4*. The Chr. 2A retrocopy has multiple internal start and stop sites, resulting in potentially smaller peptides. This results in one putative peptide that resembles *ATF4P1/2* Small X, but none that contain as much of the p300 domain as *ATF4P1/2* Long X. The retrocopy on Chr. 15 lacks the p300 domain due to a chromosomal truncation of DNA (unlike the previous protein-level truncations). The retrocopy on Chr. 17 may be a duplication of the ATF4P4-like retrocopy because it is only ~1 Mb away. In addition, it is only a truncated copy of this gene with only one flanking tandem duplication site (TDS) rather than the two sites expected if it were another LINE-1 element-derived retrocopy. However, it is nevertheless possible that this arose independently and later lost one original flanking TDS.

Approximate evolutionary timeline

The first retrotransposition event occurred ~37 mya (Figure 2F). Later, approximately 14.8 mya,[69–73] the common ancestor of humans, chimps, bonobos, and gorillas had three independent duplications of *ATF4*—two retroduplications (*ATF4P3*, *ATF4P4*) and one duplication of a retrocopy (*ATF4P1* duplicated from *ATF4P2*).[81] This suggests a period of stronger selection for *ATF4* retrocopies ~14.8 mya (Figure 2F). In apes, additional *ATF4* retrocopies have arisen since then in orangutans (~14.8 mya or earlier) and bonobos (~2 mya or earlier) (Figure 2F).

Independent *ATF4* retrocopies arose in many other mammals

There are many genomically intact (e.g., not heavily degraded or fragmented) retrocopies of *ATF4* throughout mammals (Tables 3 and S2). In addition to the extra retrocopies in orangutan and gibbon genomes (Tables S1 and S2; Figure S3), there are multiple non-syntenic *ATF4* retrocopies in several other primates, including the Philippine tarsier (*Carlito syrichta*, four retrocopies), the mouse lemur (*Microcebus murinus*, one retrocopy), the bush baby (*Otolemur garnetti*, three retrocopies), and the flying lemur (*Galeopterus variegatus*, three retrocopies) (Table 3; Figure S3; Table S2). As none of these retrocopies are syntenic, there are at least 12 additional independent retroduplications of *ATF4* in primates, bringing the total we observed to 18 (Figure S3).

We also found evidence of independent retrocopies of *ATF4* in 13 other mammals (Table 3; Figure S3; Table S2). Most of these species only had a single retrocopy of *ATF4*, with the exception of the European hedgehog (*Erinaceus europaeus*, three retrocopies) and the nine-banded armadillo (*Dasypus novemcinctus*, four retrocopies). Of note, the Bovidae family has a syntenic retroduplication of *ATF4* present in at least bison, cows, and sheep (*Bison bison*, *Bos taurus*, and *Ovis aries*) (Figure S3; Table S2). This retrocopy is inserted within an intron of the *BANK1* gene (MIM: 610292),[3] so it may utilize its nearby regulatory sequences (albeit, the retrocopy is truncated). We see no evidence of *ATF4* retrocopies in birds, lizards, amphibians, fish, or any invertebrate species (using invertebrate orthologs; Table S2). This is unsurprising, as there are much lower rates of retrocopies in non-mammalian species.[90,91]

Likelihood of retaining ATF4 retrocopies over time

There are an estimated ~8,000 retrocopies in humans.[90,92] Because many retrocopies arise with no nearby regulatory components, with only <20% having an intact ORF, it is not uncommon for neutral evolution to eventually degrade retrocopies.[90,92] Supporting this, we found several *ATF4* retrocopies in vertebrates that appear to have degraded over time (Table S2). In contrast, persistence of an intact gene over time can suggest non-neutral evolution of that gene. *ATF4P1–4* are relatively evolutionarily young (within ~37.5 my, for example, instead of a 90+ my divergence between humans and mice[93]). This is also amplified by the long generation times in humans (~25 years) and monkeys (~10 years). Thus, it is possible that not enough time has passed to determine the likelihood of neutral evolution. However, given the multiple intact or conserved *ATF4* retrocopies in multiple ape and monkey branches, we wanted to determine the likelihood of *ATF4P1–P4* ORFs persisting over time to date. First, we determined the ratio of non-synonymous to synonymous substitution rates (dN/dS) for each retrocopy. We compared each human retrocopy to the parent gene, ATF4 (Figure S5A). All retrocopy ratios fell between 0.4 and 0.6, indicating moderate negative selection. To better

Table 3. Summary table of mammalian *ATF4* retrocopies

Common name	Species name	Total retrocopies
Human	*Homo sapiens*	4
Chimp	*Pan troglodytes*	4
Bonobo	*Pan paniscus*	4
Gorilla	*Gorilla gorilla gorilla*	4
Orangutan	*Pongo pygmaeus abelii*	5
Gibbon	*Nomascus leucogenys*	2
Green monkey	*Chlorocebus sabaeus*	1
Crab-eating macaque	*Macaca fascicularis*	1
Rhesus macaque	*Macaca mulatta*	1
Baboon (hamadryas)	*Papio hamadryas*	1
Proboscis monkey	*Nasalis larvatus*	1
Golden snub-nosed monkey	*Rhinopithecus roxellana*	1
Tarsier	*Tarsius syrichta* (*C. syrichta*)	4
Mouse lemur	*M. murinus*	1
Bush baby	*O. garnettii*	3
Malayan flying lemur	*G. variegatus*	3
Chinese hamster	*Cricetulus griseus*	1
Guinea pig	*Cavia porcellus*	1
Squirrel	*Spermophilus tridecemlineatus* (*Ictidomys tridecemlineatus*)	1
Tree shrew	*Tupaia belangeri*	1
Bison	*B. bison*	1
Cow	*B. taurus*	1
Sheep	*O. aries*	1
Pig	*Sus scrofa*	1
Megabat	*Pteropus vampyrus*	1
Dog	*Canis lupus familiaris*	1
Hedgehog	*E. europaeus*	3
Tenrec	*Echinops telfairi*	1
Armadillo	*D. novemcinctus*	4

Mammals in which at least one full-length or truncated retrocopy were found. See Table S2 for details.

match the ancestral version of ATF4 at the time of retrocopy emergence in primates, we created approximate ancestral versions of ATF4 for each retrocopy based on likely time of emergence (Table S3). Using these versions, the dN/dS was similar in *ATF4P1/2* and *ATF4P4* (~0.4), while rising to ~0.7 in *ATF4P3* (Figure S5B). Overall, these dN/dS generally suggest negative selection on *ATF4* retrocopies.

Next, we wanted to computationally model the likelihood of ATF4P-1P4 persistence. We used the same ancestral reconstructions of *ATF4P1–P4*, as in the dN/dS analysis, to model mutations over time (Table S3). We then modeled the likelihood of maintaining the size of each retrocopy's longest ORF over 10,000 simulations of decay within each species. Both *ATF4P1/2* and *ATF4P4* have truncated ORFs, and this short size makes them statistically more likely to avoid degradation. For *ATF4P1/2*, there was an approximately 45% chance of its Long X ORF remaining intact among apes and monkeys (Figure S6A). For *ATF4P4*, with its much shorter evolutionary time, there was approximately a 97.6% chance of remaining intact (Figure S6B). *ATF4P3*, on the other hand, is completely intact after the same evolutionary time period. As such, our model found approximately only a 20% chance of remaining intact within apes (Figure S6B). While evolutionarily young, the chances

Table 4. GTEx expression metrics in each tissue type

	ATF4	*ATF4P1*	*ATF4P2*	*ATF4P3*	*ATF4P4*
# ≥0.1 TPM	52	0	0	52	49
# ≥0.5 TPM	52	0	0	13	5
Minimum TPM	98.38	0	0	0.12	0.084
Maximum TPM	806.1	0.047	0.049	0.88	2.3
Evidence in GTEx long-read data?[94]	N/A	no	no	yes	yes

See Table S4 for details. N/A, not applicable.

do not favor keeping an intact ORF the size of *ATF4P3* in four different apes. As ATF4 is a transcription factor, we also wanted to model the likelihood of the DNA-binding domain remaining completely intact in *ATF4P3* (Figures 1C and S4). We determined the likelihood that this region remained intact with no non-synonymous changes or indels in humans and chimps. The DNA-binding domain was unchanged at the protein level approximately 12.7% of the time, indicating this is even less likely to occur (Figure S6C). This is also an underestimation, as our modeling does not take into account times when an upstream frameshift or nonsense mutation would remove the DNA-binding domain. Overall, our modeling suggests at least *ATF4P3* may be under non-neutral evolution.

Lastly, we modeled the likelihood of human *ATF4P2* and *ATF4P1* remaining identical over 14.8 my. As mentioned, this area of the genome is highly invariant.[81] As such, it is possible they are genetically linked to a different gene under non-neutral evolution or have undergone interlocus gene conversion.[86] Nevertheless, it is interesting to model because, as mentioned, unlike humans, the three other apes we examined do have changes between their *ATF4P2* and *ATF4P1* retrocopies (Figures 2A and 2B [chimps, bonobos, gorillas]; Table S1). We used the same simulation as above, but we instead determined how many times there were zero changes at the nucleotide level. After modeling just ∼10 my, all 10,000 simulated replicates had at least 1 nt change (Figure S6D). As this duplication likely occurred ∼14.8 mya,[69–73] this means there is a <0.01% chance of *ATF4P2* having no changes.

ATF4 retrocopies are expressed in tissues and human cells

To determine whether the *ATF4* retrocopies are expressed in humans, we first queried the GTEx online portal for mRNA expression data of each retrocopy based on thousands of healthy individuals. *ATF4* has median transcript per million (TPM) values ranging from 98 to 806 depending on tissue (Tables 4 and S4). In contrast, *ATF4P2* and *ATF4P1* have a maximum TPM value of 0.05, and the highest *ATF4P3* and *ATF4P4* median TPM values are 0.88 and 2.3, respectively (Table 4). GTEx uses a TPM cutoff of 0.1 to be considered expressed for use in their eQTL analysis,[95] and the EMBL-EBI Expression Atlas sets a TPM cutoff of 0.5 for a gene to be considered expressed.[96,97] *ATF4P3* has a TPM of ≥0.1 in all tissues, with 13 at ≥0.5 TPM. *ATF4P4* has a TPM of ≥0.1 in 49/52 tissues, with five at ≥0.5 TPM. However, *ATF4P2* and *ATF4P1* never reach these thresholds. Long-read sequencing may better resolve these four highly similar retrocopy transcripts, so we also queried GTEx Oxford Nanopore Technology (ONT) data for these ATF4 retrocopies.[94] We found evidence for *ATF4P3* and *ATF4P4* in these long-read data, but we could not find *ATF4P2* or *ATF4P1*. However, because *ATF4P2* and *ATF4P1* have 100% identical transcripts, their inability to be uniquely identified might cause analysis pipelines to exclude them. Interestingly, we also queried the ONT data for several known retrogenes, *PTENP1*,[37,38] *PGK2*,[41,98] and *HAPSTR2*,[35] and could only find transcripts of *HAPSTR2*. Thus, these long-read data do not definitively rule out *ATF4P1/2* expression but they at least lend more credence to *ATF4P3* and *ATF4P4* expression. Overall, this suggests a low level of documented basal expression for *ATF4P3* and *ATF4P4* with none for *ATF4P2* and *ATF4P1*.

We next examined transcriptional markers around each retrocopy by using psiCube, which analyzes pseudogenes across humans, flies, and worms.[99] Based on psiCube annotation, both *ATF4P3* and *ATF4P4* have active chromatin (pseudogene activity resource, psiCube, April 2014[99]). We examined this further by querying ENCODE data from seven human cell lines for chromatin markers.[100] *ATF4P1*, *ATF4P2*, and *ATF4P3* have H3K4Me1 markers, indicative of enhancer elements,[101] nearby their insertion sites (Figure S7A). *ATF4P4* has a H3K27Ac marker, also indicative of enhancer elements[102] (Figure S7A). *ATF4P3* and *ATF4P4* each harbor H3K4Me3 markers, which are often found near transcription start sites[103] (Figure S7A). We also note that *ATF4P4* is inserted directly next to a different retrocopy, *SH3GL1* (MIM: 601768),[3] but the *SH3GL1* retrocopy has no H3K4Me3 markers (Figure S7A), indicating specificity to *ATF4P4*. Adding to these chromatin data, we examined cCREs from ENCODE data.[52] ATF4P3 and ATF4P4 have several cCRE markers indicating potential regulatory elements and open chromatin (Figure S7B). We next looked at transcription start site (TSS) mapping data (through Cap Analysis of Gene Expression [CAGE] sources),[53] and there are

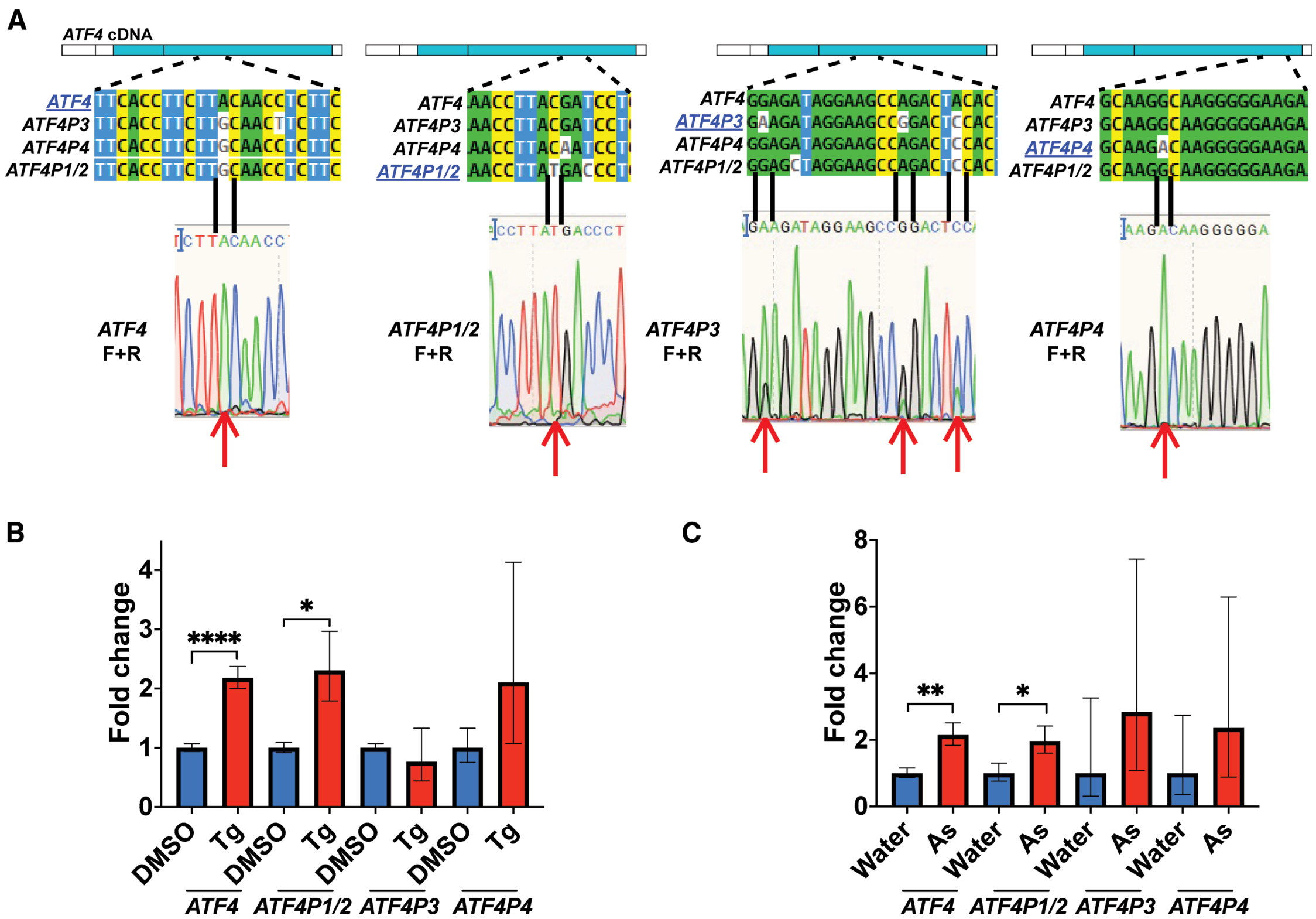

Figure 3. Chromatogram analysis identifies endogenous *ATF4* retrocopy transcripts in human cells
(A) We used primers specific to each gene to amplify transcripts under normal conditions. Using SNPs, we identified sections of each amplified transcript that differed between each gene. Black bars denote the SNPs used, and the transcript being examined is in blue. We checked the chromatograms of these regions to determine how clean each transcript signal was (e.g., whether *ATF4*, or other retrocopies, were being partially amplified). *ATF4*, *ATF4P1/2*, and *ATF4P4* had clean peaks, while *ATF4P3* had slight contamination from *ATF4* transcripts. Overall, we were able to detect that every retrocopy transcript was present in cells. We created alignments using ClustalOmega and visualized them using MView.[61]
(B) Using the primers from (A), thapsigargin (Tg) treatment induced *ATF4* and *ATF4P1/2* expression ($n = 5$ biological replicates; ****$p < 0.0001$, *$p < 0.05$, Student's *t* test).
(C) Like Tg treatment, treatment with arsenite (As) induced *ATF4* and *ATF4P1/2* expression ($n = 3$ biological replicates; **$p < 0.01$, *$p < 0.05$, Student's *t* test). For (B) and (C), we plot the average fold change and the standard error.

strong TSS signals in *ATF4P3*, moderate signals in *ATF4P1* and *ATF4P4*, and only weak signals in *ATF4P2* (Figure S7B). Finally, given their connection to *ATF4* and stress, we examined retrocopies for JASPAR data[54] of any ISR or UPR-specific transcription factor motifs. All retrocopies have a nearby (<1 kb) ATF3-binding motif (Figure S7B), which is a target of ATF4.[104,105] *ATF4P1/2* and *ATF4P3* have motifs of either CEBPA (upregulated by the ISR[106]) or CEBPA dimerized with DDIT3/CHOP (albeit further away) (Figure S7B). Finally, *ATF4P1/2* also have an XBP1-binding motif that is associated with the UPR (Figure S7B). Taken together, these regulatory elements agree with GTEx expression data showing evidence for expression of *ATF4P3* and *ATF4P4* and weak or no expression of *ATF4P1/2*.

To verify the mRNA expression of these retrocopies, we tested endogenous retrocopy expression levels in a human cell line. Because *ATF4* interacts with tumor cell biology,[8,12–17] we avoided using common cancer-derived cell lines such as HeLa cells. Thus, we used hTERT RPE-1 cells, as they are a noncancerous human cell line that is karyotypically normal (CRL-4000, ATCC).[107,108] The *ATF4* retrocopies are highly identical to *ATF4* (Figure 1B), so we designed primers to minimize off-target binding to each other. As the 3′ end of each primer is most important for binding and subsequent synthesis by polymerase,[56] we designed primers with a mismatch at the 3′ end of either the forward or reverse primer (see materials and methods). For *ATF4P1/P2*, we instead designed a primer around a large deletion only present in *ATF4P1/2*. Each ∼100- to 200-bp product contained single-nucleotide variants (SNVs) with which we verified amplification of the correct cDNA. Importantly, we used DNAse treatment to eliminate genomic DNA contamination from our RNA samples, and we verified the efficacy of this treatment (Figure S8A). Using these primers and methodology, we sequenced RNA-derived cDNA from WT RPE-1 cells.

Using Sanger sequencing and chromatogram analysis, we successfully identified expression of each specific retrocopy of *ATF4* (Figure 3A). *ATF4P1/P2* and *ATF4P4* produced clean SNVs distinguishing them from both *ATF4* and the other *ATF4* retrocopies (Figure 3A). However,

ATF4P3 was partially contaminated by *ATF4*, likely due to its high similarity to *ATF4* (>95%) preventing improvements to primer design. Nevertheless, we identified distinct *ATF4P3* transcripts via *ATF4P3*-specific nucleotide changes and lack of contamination by *ATF4P4* and *ATF4P1/2* (Figures 3A and S8B). Of note, we find that *ATF4P1/2* is indeed transcribed, despite its low expression and absence of long-read sequences, on GTEx (Table 4). Overall, we find that all *ATF4* retrocopies are endogenously transcribed in RPE-1 human cells.

ATF4 expression is transcriptionally and translationally induced by multiple inputs into the ISR.[1] We tested whether inducing the ISR also induced mRNA expression of *ATF4* retrocopies. We exposed WT RPE-1 cells to thapsigargin (Tg), a SERCA inhibitor that causes ER stress and activates the PERK branch of the ISR.[1,109] As previously shown,[110–112] Tg induces mRNA expression of *ATF4* (Figure 3B). Remarkably, Tg also increased mRNA expression of the retrocopy *ATF4P1/2* to approximately the same fold increase (~2-fold). Tg did not significantly increase *ATF4P3* or *ATF4P4*. To determine whether the *ATF4P1/2* increase in expression was from ISR induction and not a Tg-specific response, we tested a second inducer of the ISR. We exposed cells to sodium As—an inorganic compound that activates the HRI branch of the ISR.[1,113] As with Tg, As induced mRNA expression of both *ATF4* and *ATF4P1/2* (Figure 3C), but it did not significantly increase *ATF4P3* or *ATF4P4*. While neither ISR-inducing drug significantly increased *ATF4P3* or *ATF4P4* expression, there was high variably across both drugs tested (Table S5). It is possible that more stable induction might be achieved using more precise timing or dosage of ISR-inducing drugs.

ATF4 retrocopies can be translated and are degraded by the proteasome

To determine the effects of these retrocopies in human cells, we used lentiviral transfection to create stable OE lines of *ATF4* and each of the *ATF4* retrocopy ORFs in RPE-1 cells. We created each OE cell line and confirmed correct sequencing—including the presence of an in-frame V5 tag. Using the V5 tag, we verified correct band sizes for each of the overexpressed proteins except for the ATF4P1/2 Small X retrocopy, from which we were unable to verify protein on our western blots (likely due to its small size) (Figures S9A and S9B). These OE cell lines had no gross defects when compared to WT cells.

As described, ATF4 has two degradation domains: (1) an ODD domain and (2) a βTrCP motif. Each of these domains result in the sequestration to, and termination at, the proteasome.[1,20,24] As such, inhibiting the proteasome stabilizes ATF4 protein levels,[24,114] so we tested whether that is also true for ATF4 retrocopies. To inhibit the proteasome, we used Btz.[114,115] As expected, ATF4 protein increased under Btz treatment (Figures 4A, S9B, and S9C). In each retrocopy OE cell line, the protein was also increased under proteasome inhibition (Figures 4A, S9B, and S9C), indicating that these are also degraded by the proteasome.

Surprisingly, while ATF4P4 and ATF4P1/2 lack both of the ATF4 degradation domains (Figure 1C), they still showed increased protein levels with proteasome inhibition. Given the lack of these domains, it is likely that a different mechanism underlies their eventual degradation by the proteasome. We also observed a second band at ~10–12 kDa in the ATF4P4 OE line (Figures S9A–S9C) that also increased under Btz treatment. We note that there is a second methionine start site within ATF4P4, which, if translated, would be approximately the observed size (annotated in Figure S9D). Finally, we also note that ATF4P4 and ATF4P1/2 have a stronger relative response to Btz treatment (Figures 4A, S9B, and S9C). This could suggest that they have a faster turnover rate by the proteasome. However, the proteasome must unfold ubiquitinated proteins prior to processing.[116] Given that ATF4P4 and ATF4P1/2 are both truncated, they may simply have a less stable folded product and are thereby more easily processed by the proteasome—regardless of any particular regulatory mechanism. Taken together, all ATF4 retrocopy proteins are degraded by the proteasome.

ATF4 retrocopies affect expression of canonical *ATF4* target genes

ATF4 is a transcription factor that affects the expression of hundreds of genes.[5–7] We wanted to determine what effect, if any, ATF4P1–4 has on the expression of a diverse set of ATF4 target genes. We selected five ATF4 transcriptional target genes relating to unique downstream pathways: *TRIB3* (MIM: 607898),[3] involved in negative feedback of ATF4[1]; *ATF3* (MIM: 603148),[3] a transcription factor important for stress and regeneration[1]; *SARS1* (MIM: 607529),[3] a tRNA charging protein[6]; *XPOT* (MIM: 603180),[3] involved in export of tRNAs[6]; and *ERO1A*, involved in protein folding.[6] We determined the expression of these genes under ATF4 and retrocopy OE. To simulate cell stress resulting in increased expression of these proteins, we also determined gene expression under Btz treatment. Note that we included our nucleotide sequence-verified ATF4P1/2 Small X cell line in this assay, although we could not verify the expression of its small peptide (Figure S9B).

ATF4P3 increased *XPOT* expression, with or without Btz treatment (Figure 4B). It also increased *SARS1* expression without Btz and *ERO1A* expression with Btz, but neither to a statistically significant level. As ATF4P3 most closely resembles ATF4, we expected that this protein would be most able to increase expression of ATF4 target genes. However, its effect on increasing gene expression was smaller compared to the other retrocopies—suggesting either that its high identity to ATF4 means it is also tightly controlled[1] or that one or more of its 15 single-amino acid variants (Figure 1C) affect its ability to alter gene expression.

ATF4P4 had the strongest overall effect on the expression of ATF4 target genes, but, surprisingly, it decreased

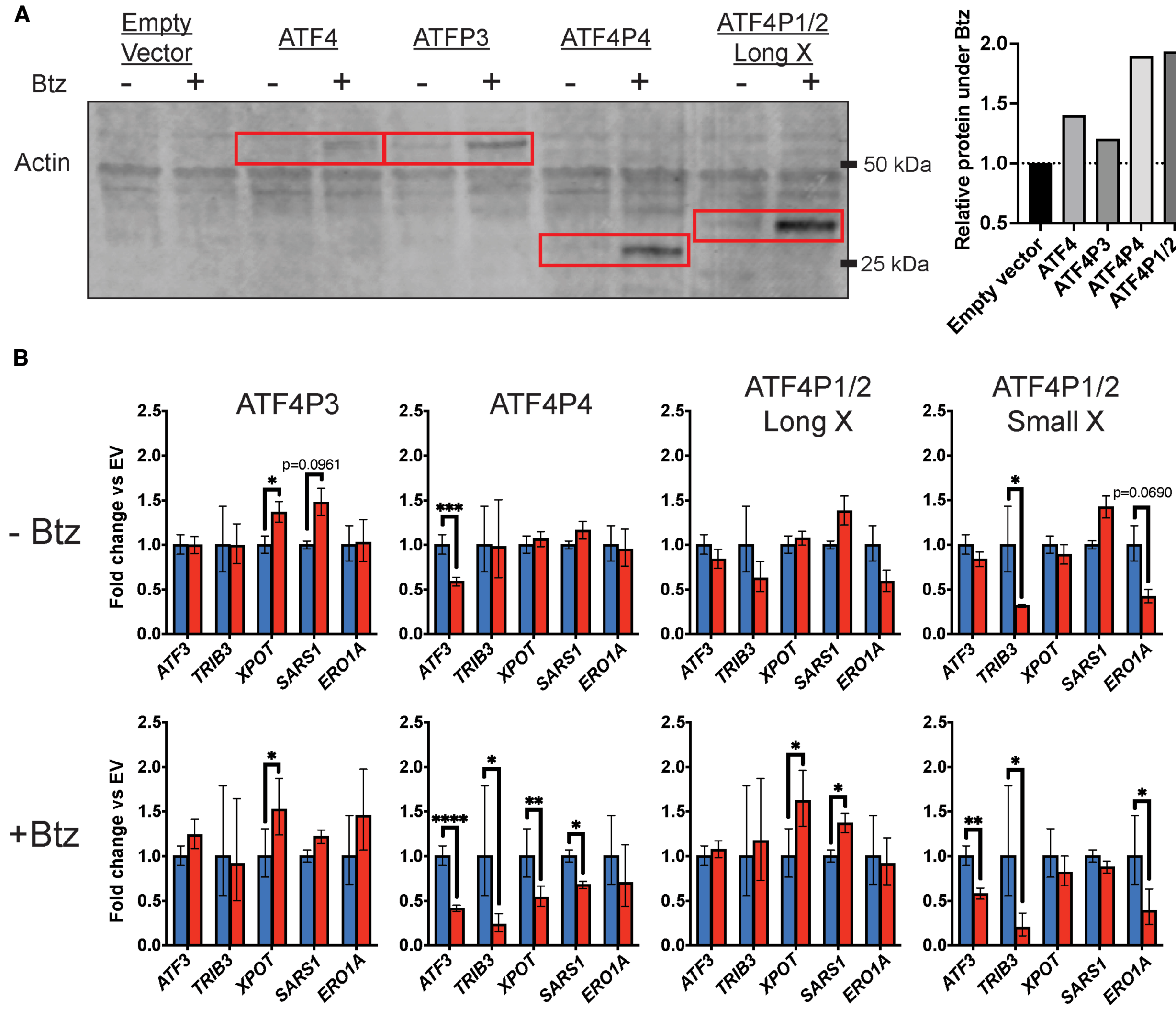

Figure 4. *ATF4* retrocopy proteins are degraded by the proteasome and affect the expression of canonical *ATF4* gene targets

(A) We grew each cell line overexpressing *ATF4* and its retrocopies with or without proteasome inhibition (through Btz). Proteasome inhibition increased the ratio of *ATF4*/retrocopy protein in each instance (see red boxes and rightmost graph). Note that image contrast was globally adjusted for band clarity; see full gel in Figure S9.

(B) We analyzed gene expression of five known *ATF4* target genes in each OE cell line, with or without proteasome inhibition. Blue bars represent empty vector (EV) control cell lines, and red bars represent cell lines overexpressing the gene indicated above each graph. Generally, the increased levels of each retrocopy protein from proteasome inhibition (in A) had a stronger impact on the expression of each *ATF4* target gene. However, there were gene expression changes under both conditions, indicating that these retrocopies can impact *ATF4* gene expression ($n = 3$ biological replicates; ****$p < 0.0001$, ***$p < 0.001$, **$p < 0.01$, *$p < 0.05$, one-way ANOVA with adjusted p values derived using Dunnett's multiple comparison correction).

expression of those genes rather than increased them (Figure 4B). ATF4P4 significantly reduced the expression of *ATF3* with or without Btz. ATF4P4 also reduced the expression of *TRIB3*, *XPOT*, and *SARS1*, but only with Btz treatment. ATF4P4 lacks the basic leucine zipper domain, including its internal nuclear localization signal (Figure 1C), so this is unlikely due to any DNA-binding activity. The truncated form of ATF4P4 leaves only the p300-binding domain intact (Figure 1C). Thus, one potential mechanism is that ATF4P4 competes with ATF4 for p300 binding, thereby reducing the stability of ATF4 and its transcription of DNA.[19] Alternatively, ATF4P4 may bind ATF4 co-binding proteins, such as CHOP,[18] to prevent formation of heterodimers necessary for gene transcription. Overall, ATF4P4 expression negatively impacts the expression of several canonical ATF4 target genes.

While ATF4P1/2 Long X has a similarly truncated protein like ATF4P4 (Figure 1C), it had a smaller effect on gene expression (Figure 4B). Under Btz treatment, ATF4P1/2 Long X increased both *XPOT* and *SARS1* expression. However, ATF4P1/2 Long X lacks the first 16 aa of the p300-binding domain, which may weaken any potential binding to p300 and any subsequent effects on ATF4. This is further supported by the fact that the ATF4P1/2 Small X peptide, which is simply the first 15 aa of the p300-binding domain (with one change, D15A)

(Figure 1C), mimics ATF4P4 by also having a negative effect on ATF4 target gene expression. With or without Btz treatment, ATF4P1/2 Small X reduced *TRIB3* expression (Figure 4B). Under Btz treatment, ATF4P1/2 Small X reduced the expression of *ATF3* and *ERO1A*. ATF4P1/2 Small X also reduced expression of *ERO1A* without Btz treatment, but not to a statistically significant degree. Given these results and the amino acid overlap of ATF4P4 and ATF4P1/2 Small X, it is possible that the first 15 aa of the p300 interaction domain have a negative effect on ATF4 target gene expression.

Overall, it is clear that, with or without cell stress, ATF4 retrocopies can alter ATF4 target gene expression. Given this, their similar protein-degradation patterns (Figure 4), their ability to be upregulated by the ISR (Figure 3), and their expression in humans (Figure 3), we conclude that *ATF4P1–4* are functional retrogenes.

Discussion

We characterized four retrogenes of the stress transcription factor *ATF4* in humans. We found these retrogenes across multiple primate lineages, suggesting an increase in their selection ~14.8 mya. These retrogenes are expressed at the mRNA level in cells—including *ATF4P1/2*, which responds to induction of the ISR. Overexpressing stable proteins of *ATF4* retrocopies alters the expression of *ATF4* target genes, indicating their potential for biological function. Given the important role for ATF4 in human diseases, such as cancer,[8,12–17] our findings suggest that these *ATF4* retrogenes should be considered when studying the ISR and ATF4 pathways.

The increase in LINE-1 element transposons in mammalian lineages resulted in an increase in their total retrocopied genes.[90,117] Humans have ~8,000 retrocopies, but most remain uncharacterized or unstudied.[34,90,92,118] However, there are several instances of retrocopies having biological function. HAPSTR2 is a retrogene that aids in cell stress signaling.[35] The retrogene *retroCHMP3* inhibits viral budding.[36,76] The testis-specific retrogene *PGK2* positively regulates sperm function.[41,98] At the mRNA level, the retrogene *PTENP1* regulates PTEN expression at the mRNA level.[37,38] Its transcript acts as a decoy that pulls inhibiting microRNAs away from PTEN, thereby increasing its expression.[37,38] Thus, *ATF4P1–4* are yet another example of retrogenes capable of affecting cell biology. Of note, retrogenes can interact with their parent gene and/or evolve new function. For example, *HAPSTR2* increases the stability of its parent gene, *HAPSTR1*, but it also impacts stress signaling when *HAPSTR1* is knocked out.[35] On the other hand, the function of *PTENP1* appears to primarily revolve around changing expression of its parent gene, *PTENP1*, without having independent function. While the ATF4 retrocopies can impact expression of known ATF4 target genes, it is still unclear whether they also have independent functionality. Neofunctionalization in stress transcription factor families is not uncommon. For example, *ATF*s have seven members (*ATF1–7*), which share similar DNA-binding domains and recognition sequences but which have different target genes and expression profiles.[119] Similarly, HSF transcription factors have also diversified into having several specialized functions, likely through duplication events.[120] Thus, it will be worth testing the ATF4 retrocopies as to whether they have also gained any neofunctionality.

Approximately 30% of retrocopies have RNA expression data.[117,121] However, previous expression data must be validated. For example, there is evidence of *ATF4P1/2* mRNA expression in our cells, yet *ATF4P1/2* does not have significant expression in GTEx.[122] In addition, while we find evidence for *ATF4P3* and *ATF4P4* in GTEx long-read data, we did not find *ATF4P1* and *ATF4P2*. In this case, this might be due to the unique genetic region containing *ATF4P1/2*; however, we also did not find long-read transcripts for two other retrogenes (*PTENP1* and *PGK2*) despite these having established expression.[37,38,41,98] Therefore, individual retrocopy expression should be more closely examined on a case-by-case basis, and it is possible that some retrocopy transcripts require transcript-specific primers to distinguish them.

When making OE constructs, we were surprised that adding endogenous *ATF4*, or retrocopy, CDSs driven by a CMV promoter did not produce protein (tested via western blot, in both transfected RPE-1 and HeLa cells). Adding the entire 5′ UTR with the CDS also did not produce protein. Only by editing the Kozak sequence (see materials and methods) upstream of the ATG were we able to resolve protein. ATF4 protein is translated basally, even in the absence of stress,[6] and *ATF4P3* and *ATF4P4* share the same Kozak sequence. Thus, while we cannot rule out that endogenous translation of ATF4P3 or ATF4P4 might be affected by this Kozak sequence, at least they would likely be affected to the same degree as parental ATF4. However, the *ATF4P1/2* Small X has an SNP in its Kozak sequence, so it is possible that its endogenous translation could be affected.

The ISR is a critical stress-response pathway conserved from yeast to humans.[1,123] It is crucial for restoring cell homeostasis under various stressors.[1] It is dysregulated in some individuals with Wolcott-Rallison syndrome[124] (MIM: 226980)[3] and pulmonary disease,[125] as well as in several other disease models.[126–128] We found that the retrogenes *ATF4P1/2* are upregulated under the ISR stressors As and Tg[113,129] (Figure 3). In addition, OE of *ATF4P1/2* altered expression of several canonical *ATF4* target genes (Figure 4)—positively or negatively depending on the ORF involved. he *ATF4P1/2* retrogene is the oldest retrocopy in humans (Figure 2), and both copies remain unchanged at the nucleotide level for ~14 my in humans. Thus, *ATF4P1/2* may actively participate in the ISR in humans and should be considered when further studying these pathways and disease models. *ATF4P3* and *ATF4P4* had more variable expression under ISR stress (Figure 3),

and this may indicate they need different conditions for their expression. For example, *ATF4P3* is inserted in the intron of *RNF157* (Figure S2), which has strong brain expression[130] (and also via GTEx[122]). Thus, it may require a neuronal model or cell line to fully capture the expression or effects of *ATF4P3*.

Retrocopies can affect the expression of their parent genes at the transcriptional or translational level.[34,36–38,121] Based on our proteasome inhibition experiments (Figure 4), all four *ATF4* retrogene proteins are typically degraded by the proteasome (although not necessarily at the same rate). Inhibiting the proteasome, and thus increasing their protein levels, alters the expression of ATF4 target genes. This suggests that their actual translated peptides can influence gene expression. While this could be explained by an intact DNA-binding domain in ATF4P3, it does not explain ATF4P4 or ATF4P1/2, which lack this domain. ATF4 typically forms a heterodimer, such as with CHOP or ATF3,[18] to bind to DNA. It is possible that ATF4P4 or ATF4P1/2 can bind—even partially—to these co-binding partners and sequester them away from ATF4. This could reduce gene expression overall or force *ATF4* to bind with different co-binding partners and switch which genes are upregulated. Alternatively, it is possible that these retrogenes act at the RNA level (like retrogene PTENP1[37,38])—as our OE clones would presumably have increased mRNA as well—or a combination of the two. The exact mechanism of how these retrogenes influence *ATF4*, and its transcriptional ability, will be of interest to further characterize how they impact the ISR and related diseases.

As noted above, we find a second band at ~10–12 kDa in the ATF4P4 OE line (Figures S9A–S9C) and a second methionine start site within ATF4P4 (annotated in Figure S9D). Because this smaller product of ATF4P4 also increased under Btz treatment, it is likely also degraded by the proteasome. As such, we looked within this product for potential lysine residues with evidence of ubiquitination in ATF4 (using Uniprot[49]: P18848). ATF4 residue K88 has high confidence as a ubiquitinated site,[49,131] and it is maintained in ATF4P4 and ATF4P1/2 Long X (Table S1). Thus, we hypothesize that ubiquitination of residue K88 might affect its proteasomal degradation, as it would explain why ATF4P4 and ATF4P1/2 have higher abundance under Btz treatment despite lacking the ODD domain and βTrCP motif.

While they lack their DNA-binding domain, ATF4P4 and ATF4P1/2 maintain their p300 interaction domain (Figure 1C). As p300 usually stabilizes ATF4 and helps prevent its degradation,[19] it is possible that ATF4P4 and ATF4P1/2 sequester p300 away from ATF4 and thereby reduce its expression. Additionally, the ability to bind p300 offers an explanation for the evolutionary origin of these retrogenes, including our finding that humans, chimps, bonobos, and gorillas have at least two "p300-only" retrocopies. Certain viruses can hijack p300 and use it to aid the transcription of viral machinery.[132–134] For example, the HIV-1 Tat protein is acetylated by p300 to aid its transcription,[135] and adenovirus E1A uses p300 to modulate gene expression.[136] Outside of any effect on ATF4 target gene expression, it is possible that ATF4P4 and ATF4P1/2 sequester p300 away from viruses to help limit viral gene transcription. Given that the ISR is known to have evolutionary pressure from viruses,[42,43] this provides a potential source of evolutionary pressure and selection to help explain the presence of maintained, but truncated, *ATF4* retrogenes.

Data and code availability

Data/sequences generated or organized in this study are contained within the figures, tables, and supplementary files with links to original databases alongside them when applicable. The majority of code used in this study was derived from the literature or public sources (cited when used). Our modified code of the mutator script[62,63] is available on GitHub (https://github.com/hansmdalton/pedestal_fork).

Acknowledgments

We thank Erik Eide and Dr. Charles Murtaugh for their help in lentiviral cloning, western blots, and use of equipment. We thank Dr. Nels Elde and Diane Downhour for their samples and guidance in retrocopy experiments and strategies. We thank the HSC DNA Sequencing Core (director Dr. Derek Warner) at the University of Utah for their help in sequencing analysis. We thank Mr. Ben Anderson for his help in manually collecting GTEx data. This work was supported by National Institute of General Medical Sciences (NIGMS, www.nigms.nih.gov/) R35GM124780 to C.Y.C. It was also supported by NIGMS F32GM136057 and Eunice Kennedy Shriver National Institute of Child Health and Human Development (NICHD, https://www.nichd.nih.gov/) K99HD111662/R00HD111662 to H.M.D. The funders had no role in study design, data collection and analysis, decision to publish, or preparation of the manuscript.

Declaration of interests

The authors declare no competing interests.

Supplemental information

Supplemental information can be found online at https://doi.org/10.1016/j.xhgg.2026.100666.

Web resources

Clustal Omega[55], https://www.ebi.ac.uk/jdispatcher/msa/clustalo

Ensembl[48], https://www.ensembl.org/

Expasy Translation Tool[137], https://web.expasy.org/translate/

GTEx Portal[122], https://www.gtexportal.org/

Interactive Tree of Life[51], https://itol.embl.de/

Online Mendelian Inheritance in Man[3], https://www.omim.org/

PrimerBank[57], https://pga.mgh.harvard.edu/primerbank/

UCSC Genome Browser[138], https://genome.ucsc.edu/cgi-bin/hgGateway

Received: April 23, 2026
Accepted: August 24, 2026

References

1. Pakos-Zebrucka, K., Koryga, I., Mnich, K., Ljujic, M., Samali, A., and Gorman, A.M. (2016). The integrated stress response. EMBO Rep. *17*, 1374–1395. https://doi.org/10.15252/embr.201642195.
2. Ryoo, H.D. (2024). The integrated stress response in metabolic adaptation. J. Biol. Chem. *300*, 107151. https://doi.org/10.1016/j.jbc.2024.107151.
3. Online Mendelian Inheritance in Man, OMIM®. McKusick-Nathans Institute of Genetic Medicine, Johns Hopkins University. https://www.omim.org/.
4. Guan, B.J., van Hoef, V., Jobava, R., Elroy-Stein, O., Valasek, L.S., Cargnello, M., Gao, X.H., Krokowski, D., Merrick, W.C., Kimball, S.R., et al. (2017). A Unique ISR Program Determines Cellular Responses to Chronic Stress. Mol. Cell *68*, 885–900.e6. https://doi.org/10.1016/j.molcel.2017.11.007.
5. Luhr, M., Torgersen, M.L., Szalai, P., Hashim, A., Brech, A., Staerk, J., and Engedal, N. (2019). The kinase PERK and the transcription factor ATF4 play distinct and essential roles in autophagy resulting from tunicamycin-induced ER stress. J. Biol. Chem. *294*, 8197–8217. https://doi.org/10.1074/jbc.RA118.002829.
6. Torrence, M.E., MacArthur, M.R., Hosios, A.M., Valvezan, A.J., Asara, J.M., Mitchell, J.R., and Manning, B.D. (2021). The mtorc1-mediated activation of atf4 promotes protein and glutathione synthesis downstream of growth signals. eLife *10*, e63326. https://doi.org/10.7554/eLife.63326.
7. Örd, T., Örd, D., Adler, P., and Örd, T. (2023). Genome-wide census of ATF4 binding sites and functional profiling of trait-associated genetic variants overlapping ATF4 binding motifs. PLoS Genet. *19*, e1011014. https://doi.org/10.1371/journal.pgen.1011014.
8. Bohlen, J., Harbrecht, L., Blanco, S., Clemm von Hohenberg, K., Fenzl, K., Kramer, G., Bukau, B., and Teleman, A.A. (2020). DENR promotes translation reinitiation via ribosome recycling to drive expression of oncogenes including ATF4. Nat. Commun. *11*, 4676. https://doi.org/10.1038/s41467-020-18452-2.
9. Zhao, Y., Zhou, J., Liu, D., Dong, F., Cheng, H., Wang, W., Pang, Y., Wang, Y., Mu, X., Ni, Y., et al. (2015). ATF4 plays a pivotal role in the development of functional hematopoietic stem cells in mouse fetal liver. Blood *126*, 2383–2391. https://doi.org/10.1182/blood-2015-03-633354.
10. Wang, W., Lian, N., Li, L., Moss, H.E., Wang, W., Perrien, D.S., Elefteriou, F., and Yang, X. (2009). Atf4 regulates chondrocyte proliferation and differentiation during endochondral ossification by activating Ihh transcription. Development *136*, 4143–4153. https://doi.org/10.1242/dev.043281.
11. Tanaka, T., Tsujimura, T., Takeda, K., Sugihara, A., Maekawa, A., Terada, N., Yoshida, N., and Akira, S. (1998). Targeted disruption of ATF4 discloses its essential role in the formation of eye lens fibres. Genes Cells *3*, 801–810. https://doi.org/10.1046/j.1365-2443.1998.00230.x.
12. Ye, J., Kumanova, M., Hart, L.S., Sloane, K., Zhang, H., De Panis, D.N., Bobrovnikova-Marjon, E., Diehl, J.A., Ron, D., and Koumenis, C. (2010). The GCN2-ATF4 pathway is critical for tumour cell survival and proliferation in response to nutrient deprivation. EMBO J. *29*, 2082–2096. https://doi.org/10.1038/emboj.2010.81.
13. Pällmann, N., Livgård, M., Tesikova, M., Zeynep Nenseth, H., Akkus, E., Sikkeland, J., Jin, Y., Koc, D., Kuzu, O.F., Pradhan, M., et al. (2019). Regulation of the unfolded protein response through ATF4 and FAM129A in prostate cancer. Oncogene *38*, 6301–6318. https://doi.org/10.1038/s41388-019-0879-2.
14. Wortel, I.M.N., van der Meer, L.T., Kilberg, M.S., and van Leeuwen, F.N. (2017). Surviving Stress: Modulation of ATF4-Mediated Stress Responses in Normal and Malignant Cells. Trends Endocrinol. Metab. *28*, 794–806. https://doi.org/10.1016/j.tem.2017.07.003.
15. Rouschop, K.M.A., van den Beucken, T., Dubois, L., Niessen, H., Bussink, J., Savelkouls, K., Keulers, T., Mujcic, H., Landuyt, W., Voncken, J.W., et al. (2010). The unfolded protein response protects human tumor cells during hypoxia through regulation of the autophagy genes MAP1LC3B and ATG5. J. Clin. Investig. *120*, 127–141. https://doi.org/10.1172/JCI40027.
16. Chen, J., Huang, X., Zhang, S., and Zhu, X. (2024). ATF4 inhibits tumor development and mediates p-GCN2/ASNS upregulation in colon cancer. Sci. Rep. *14*, 13042. https://doi.org/10.1038/s41598-024-63895-y.
17. Lorenz, N.I., Sittig, A.C.M., Urban, H., Luger, A.L., Engel, A.L., Münch, C., Steinbach, J.P., and Ronellenfitsch, M.W. (2021). Activating transcription factor 4 mediates adaptation of human glioblastoma cells to hypoxia and temozolomide. Sci. Rep. *11*, 14161. https://doi.org/10.1038/s41598-021-93663-1.
18. Siang, S., Underbakke, E.S., and Roche, J. (2022). Intricate coupling between the transactivation and basic-leucine zipper domains governs phosphorylation of transcription factor ATF4 by casein kinase 2. J. Biol. Chem. *298*, 101633. https://doi.org/10.1016/j.jbc.2022.101633.
19. Lassot, I., Estrabaud, E., Emiliani, S., Benkirane, M., Benarous, R., and Margottin-Goguet, F. (2005). p300 modulates ATF4 stability and transcriptional activity independently of its acetyltransferase domain. J. Biol. Chem. *280*, 41537–41545. https://doi.org/10.1074/jbc.M505294200.
20. Köditz, J., Nesper, J., Wottawa, M., Stiehl, D.P., Camenisch, G., Franke, C., Myllyharju, J., Wenger, R.H., and Katschinski, D.M. (2007). Oxygen-dependent ATF-4 stability is mediated by the PHD3 oxygen sensor. Blood *110*, 3610–3617. https://doi.org/10.1182/blood-2007-06-094441.
21. Nakayama, K., Qi, J., and Ronai, Z. (2009). The ubiquitin ligase Siah2 and the hypoxia response. Mol. Cancer Res. *7*, 443–451. https://doi.org/10.1158/1541-7786.MCR-08-0458.
22. Ray, D., Osmundson, E.C., and Kiyokawa, H. (2006). Constitutive and UV-induced fibronectin degradation is a ubiquitination-dependent process controlled by β-TrCP. J. Biol. Chem. *281*, 23060–23065. https://doi.org/10.1074/jbc.M604311200.
23. Paul, D., Kales, S.C., Cornwell, J.A., Afifi, M.M., Rai, G., Zakharov, A., Simeonov, A., and Cappell, S.D. (2022). Revealing β-TrCP activity dynamics in live cells with a genetically encoded biosensor. Nat. Commun. *13*, 6364. https://doi.org/10.1038/s41467-022-33762-3.
24. Lassot, I., Ségéral, E., Berlioz-Torrent, C., Durand, H., Groussin, L., Hai, T., Benarous, R., and Margottin-Goguet, F. (2001). ATF4 Degradation Relies on a Phosphorylation-Dependent Interaction with the SCF βTrCP Ubiquitin Ligase. Mol. Cell Biol. *21*, 2192–2202. https://doi.org/10.1128/MCB.21.6.2192-2202.2001.

25. Smirnova, A.M., Hronová, V., Mohammad, M.P., Herrmannová, A., Gunišová, S., Petráčková, D., Halada, P., Coufal, Š., Świrski, M., Rendleman, J., et al. (2024). Stem-loop-induced ribosome queuing in the uORF2/ATF4 overlap fine-tunes stress-induced human ATF4 translational control. Cell Rep. *43*, 113976. https://doi.org/10.1016/j.celrep.2024.113976.
26. Vattem, K.M., and Wek, R.C. (2004). Reinitiation involving upstream ORFs regulates ATF4 mRNA translation in mammalian cells. Proc. Natl. Acad. Sci. USA *101*, 11269–11274. https://doi.org/10.1073/pnas.0400541101.
27. Lu, P.D., Harding, H.P., and Ron, D. (2004). Translation re-initiation at alternative open reading frames regulates gene expression in an integrated stress response. J. Cell Biol. *167*, 27–33. https://doi.org/10.1083/jcb.200408003.
28. Barbosa, C., Peixeiro, I., and Romão, L. (2013). Gene Expression Regulation by Upstream Open Reading Frames and Human Disease. PLoS Genet. *9*, e1003529. https://doi.org/10.1371/journal.pgen.1003529.
29. Kondrashov, F.A., Rogozin, I.B., Wolf, Y.I., and Koonin, E.V. (2002). Selection in the evolution of gene duplications. Genome Biol. *3*, RESEARCH0008. research0008.1. https://doi.org/10.1186/gb-2002-3-2-research0008.
30. Ohno, S. (1970). Evolution by Gene Duplication. Evolution by Gene Duplication (Springer Berlin Heidelberg). https://doi.org/10.1007/978-3-642-86659-3.
31. Bergthorsson, U., Andersson, D.I., and Roth, J.R. (2007). Ohno's dilemma: Evolution of new genes under continuous selection. Proc. Natl. Acad. Sci. USA *104*, 17004–17009. https://doi.org/10.1073/pnas.0707158104.
32. Esnault, C., Maestre, J., and Heidmann, T. (2000). Human LINE retrotransposons generate processed pseudogenes. Nat. Genet. *24*, 363–367. https://doi.org/10.1038/74184.
33. Richardson, S.R., Doucet, A.J., Kopera, H.C., Moldovan, J.B., Garcia-Perez, J.L., and Moran, J.V. (2015). The Influence of LINE-1 and SINE Retrotransposons on Mammalian Genomes. Microbiol. Spectr. *3*. MDNA3-0061-2014. https://doi.org/10.1128/microbiolspec.MDNA3-0061-2014.
34. Kaessmann, H., Vinckenbosch, N., and Long, M. (2009). RNA-based gene duplication: Mechanistic and evolutionary insights. Nat. Rev. Genet. *10*, 19–31. https://doi.org/10.1038/nrg2487.
35. Amici, D.R., Cingoz, H., Alasady, M.J., Alhayek, S., Phoumyvong, C.M., Sahni, N., Yi, S.S., and Mendillo, M.L. (2023). The HAPSTR2 retrogene buffers stress signaling and resilience in mammals. Nat. Commun. *14*, 152. https://doi.org/10.1038/s41467-022-35697-1.
36. Rheinemann, L., Downhour, D.M., Bredbenner, K., Mercenne, G., Davenport, K.A., Schmitt, P.T., Necessary, C.R., McCullough, J., Schmitt, A.P., Simon, S.M., et al. (2021). RetroCHMP3 blocks budding of enveloped viruses without blocking cytokinesis. Cell *184*, 5419–5431.e16. https://doi.org/10.1016/j.cell.2021.09.008.
37. Poliseno, L., Salmena, L., Zhang, J., Carver, B., Haveman, W.J., and Pandolfi, P.P. (2010). A coding-independent function of gene and pseudogene mRNAs regulates tumour biology. Nat *465*, 1033–1038. https://doi.org/10.1038/nature09144.
38. Vitiello, M., Evangelista, M., Zhang, Y., Salmena, L., Pandolfi, P.P., and Poliseno, L. (2020). PTENP1 is a ceRNA for PTEN: It's CRISPR clear. J. Hematol. Oncol. *13*, 73. https://doi.org/10.1186/s13045-020-00894-2.
39. McCarrey, J.R., and Thomas, K. (1987). Human testis-specific PGK gene lacks introns and possesses characteristics of a processed gene. Nature *326*, 501–505. https://doi.org/10.1038/326501a0.
40. Mccarrey, J.R. (1990). Molecular evolution of the human Pgk-2 retroposon. Nucleic Acids Res. *18*, 949–955. https://doi.org/10.1093/nar/18.4.949.
41. Danshina, P.V., Geyer, C.B., Dai, Q., Goulding, E.H., Willis, W.D., Kitto, G.B., McCarrey, J.R., Eddy, E.M., and O'Brien, D.A. (2010). Phosphoglycerate Kinase 2 (PGK2) Is Essential for Sperm Function and Male Fertility in Mice. Biol. Reprod. *82*, 136–145. https://doi.org/10.1095/biolreprod.109.079699.
42. Langland, J.O., Cameron, J.M., Heck, M.C., Jancovich, J.K., and Jacobs, B.L. (2006). Inhibition of PKR by RNA and DNA viruses. Virus Res. *119*, 100–110. https://doi.org/10.1016/j.virusres.2005.10.014.
43. Schoof, M., Wang, L., Cogan, J.Z., Lawrence, R.E., Boone, M., Wuerth, J.D., Frost, A., and Walter, P. (2021). Viral evasion of the integrated stress response through antagonism of eIF2-P binding to eIF2B. Nat. Commun. *12*, 7103. https://doi.org/10.1038/s41467-021-26164-4.
44. Li, J., He, J., Fu, Y., Hu, X., Sun, L.Q., Huang, Y., and Fan, X. (2017). Hepatitis B virus X protein inhibits apoptosis by modulating endoplasmic reticulum stress response. Oncotarget *8*, 96027–96034. https://doi.org/10.18632/oncotarget.21630.
45. Launer-Felty, K., Wong, C.J., and Cole, J.L. (2015). Structural analysis of adenovirus VAI RNA defines the mechanism of inhibition of PKR. Biophys. J. *108*, 748–757. https://doi.org/10.1016/j.bpj.2014.12.014.
46. Elde, N.C., Child, S.J., Geballe, A.P., and Malik, H.S. (2009). Protein kinase R reveals an evolutionary model for defeating viral mimicry. Nature *457*, 485–489. https://doi.org/10.1038/nature07529.
47. Kent, W.J. (2002). BLAT —The BLAST -Like Alignment Tool. Genome Res. *12*, 656–664. https://doi.org/10.1101/gr.229202.
48. Dyer, S.C., Austine-Orimoloye, O., Azov, A.G., Barba, M., Barnes, I., Barrera-Enriquez, V.P., Becker, A., Bennett, R., Beracochea, M., Berry, A., et al. (2025). Ensembl 2025. Nucleic Acids Res. *53*, D948–D957. https://doi.org/10.1093/nar/gkae1071.
49. UniProt Consortium (2021). UniProt: the universal protein knowledgebase in 2021. Nucleic Acids Res. *49*, D480–D489. https://doi.org/10.1093/nar/gkaa1100.
50. Abramson, J., Adler, J., Dunger, J., Evans, R., Green, T., Pritzel, A., Ronneberger, O., Willmore, L., Ballard, A.J., Bambrick, J., et al. (2024). Accurate structure prediction of biomolecular interactions with AlphaFold 3. Nature *630*, 493–500. https://doi.org/10.1038/s41586-024-07487-w.
51. Letunic, I., and Bork, P. (2024). Interactive Tree of Life (iTOL) v6: Recent updates to the phylogenetic tree display and annotation tool. Nucleic Acids Res. *52*, W78–W82. https://doi.org/10.1093/nar/gkae268.
52. Moore, J.E., Pratt, H.E., Fan, K., Phalke, N., Fisher, J., Elhajjajy, S.I., Andrews, G., Gao, M., Shedd, N., Fu, Y., et al. (2026). An expanded registry of candidate cis-regulatory elements. Nature, 1–10. https://doi.org/10.1038/s41586-025-09909-9.
53. FANTOM Consortium and the RIKEN PMI and CLST DGT, Forrest, A.R.R., Kawaji, H., Rehli, M., Baillie, J.K., de Hoon, M.J.L., Haberle, V., Lassmann, T., Kulakovskiy, I.V., Lizio,

M., et al. (2014). A promoter-level mammalian expression atlas. Nature *507*, 462–470. https://doi.org/10.1038/nature13182.
54. Ovek Baydar, D., Rauluseviciute, I., Aronsen, D.R., Blanc-Mathieu, R., Bonthuis, I., de Beukelaer, H., Ferenc, K., Jegou, A., Kumar, V., Lemma, R.B., et al. (2026). JASPAR 2026: expansion of transcription factor binding profiles and integration of deep learning models. Nucleic Acids Res. *54*, D184–D193. https://doi.org/10.1093/nar/gkaf1209.
55. Sievers, F., Wilm, A., Dineen, D., Gibson, T.J., Karplus, K., Li, W., Lopez, R., McWilliam, H., Remmert, M., Söding, J., et al. (2011). Fast, scalable generation of high-quality protein multiple sequence alignments using Clustal Omega. Mol. Syst. Biol. *7*, 539. https://doi.org/10.1038/msb.2011.75.
56. Onodera, K. (2007). Selection for 3′-End Triplets for Polymerase Chain Reaction Primers. Methods Mol. Biol. *402*, 61–74. https://doi.org/10.1007/978-1-59745-528-2_3.
57. Spandidos, A., Wang, X., Wang, H., and Seed, B. (2010). PrimerBank: A resource of human and mouse PCR primer pairs for gene expression detection and quantification. Nucleic Acids Res. *38*, D792–D799. https://doi.org/10.1093/nar/gkp1005.
58. Untergasser, A., Cutcutache, I., Koressaar, T., Ye, J., Faircloth, B.C., Remm, M., and Rozen, S.G. (2012). Primer3-new capabilities and interfaces. Nucleic Acids Res. *40*, e115. https://doi.org/10.1093/nar/gks596.
59. Wang, D., Zhang, Y., Zhang, Z., Zhu, J., and Yu, J. (2010). KaKs_Calculator 2.0: A Toolkit Incorporating Gamma-Series Methods and Sliding Window Strategies. Genomics, Proteomics Bioinforma. *8*, 77–80. https://doi.org/10.1016/S1672-0229(10)60008-3.
60. Yang, Z., and Nielsen, R. (2000). Estimating synonymous and nonsynonymous substitution rates under realistic evolutionary models. Mol. Biol. Evol. *17*, 32–43. https://doi.org/10.1093/oxfordjournals.molbev.a026236.
61. Madeira, F., Pearce, M., Tivey, A.R.N., Basutkar, P., Lee, J., Edbali, O., Madhusoodanan, N., Kolesnikov, A., and Lopez, R. (2022). Search and sequence analysis tools services from EMBL-EBI in 2022. Nucleic Acids Res. *50*, W276–W279. https://doi.org/10.1093/nar/gkac240.
62. Young, G.R., Yap, M.W., Michaux, J.R., Steppan, S.J., and Stoye, J.P. (2018). Evolutionary journey of the retroviral restriction gene Fv1. Proc. Natl. Acad. Sci. USA *115*, 10130–10135. https://doi.org/10.1073/pnas.1808516115.
63. Yang, L., Emerman, M., Malik, H.S., and McLaughlin, R.N., Jnr. (2020). Retrocopying expands the functional repertoire of APOBEC3 antiviral proteins in primates. eLife *9*, e58436. https://doi.org/10.7554/eLife.58436.
64. Campbell, C.D., and Eichler, E.E. (2013). Properties and rates of germline mutations in humans. Trends Genet. *29*, 575–584. https://doi.org/10.1016/j.tig.2013.04.005.
65. Langergraber, K.E., Prüfer, K., Rowney, C., Boesch, C., Crockford, C., Fawcett, K., Inoue, E., Inoue-Muruyama, M., Mitani, J.C., Muller, M.N., et al. (2012). Generation times in wild chimpanzees and gorillas suggest earlier divergence times in great ape and human evolution. Proc. Natl. Acad. Sci. USA *109*, 15716–15721. https://doi.org/10.1073/pnas.1211740109.
66. Xue, C., Raveendran, M., Harris, R.A., Fawcett, G.L., Liu, X., White, S., Dahdouli, M., Rio Deiros, D., Below, J.E., Salerno, W., et al. (2016). The population genomics of rhesus macaques (Macaca mulatta) based on whole-genome sequences. Genome Res. *26*, 1651–1662. https://doi.org/10.1101/gr.204255.116.
67. Rogers, J., Raveendran, M., Harris, R.A., Mailund, T., Leppälä, K., Athanasiadis, G., Schierup, M.H., Cheng, J., Munch, K., Walker, J.A., et al. (2019). The comparative genomics and complex population history of Papio baboons. Sci. Adv. *5*, eaau6947. https://doi.org/10.1126/sciadv.aau6947.
68. Pfeifer, S.P. (2017). The Demographic and Adaptive History of the African Green Monkey. Mol. Biol. Evol. *34*, 1055–1065. https://doi.org/10.1093/molbev/msx056.
69. Glazko, G.V., and Nei, M. (2003). Estimation of Divergence Times for Major Lineages of Primate Species. Mol. Biol. Evol. *20*, 424–434. https://doi.org/10.1093/molbev/msg050.
70. Springer, M.S., Meredith, R.W., Janecka, J.E., and Murphy, W.J. (2011). The historical biogeography of mammalia. Philos. Trans. R. Soc. Lond. B Biol. Sci. *366*, 2478–2502. https://doi.org/10.1098/rstb.2011.0023.
71. Ventura, M., Catacchio, C.R., Sajjadian, S., Vives, L., Sudmant, P.H., Marques-Bonet, T., Graves, T.A., Wilson, R.K., and Eichler, E.E. (2012). The evolution of African great ape subtelomeric heterochromatin and the fusion of human chromosome 2. Genome Res. *22*, 1036–1049. https://doi.org/10.1101/gr.136556.111.
72. Mitchell, J.F., and Leopold, D.A. (2015). The marmoset monkey as a model for visual neuroscience. Neurosci. Res. *93*, 20–46. https://doi.org/10.1016/j.neures.2015.01.008.
73. Yoo, D., Rhie, A., Hebbar, P., Antonacci, F., Logsdon, G.A., Solar, S.J., Antipov, D., Pickett, B.D., Safonova, Y., Montinaro, F., et al. (2025). Complete sequencing of ape genomes. Nature *641*, 401–418. https://doi.org/10.1038/s41586-025-08816-3.
74. Cock, P.J.A., Antao, T., Chang, J.T., Chapman, B.A., Cox, C.J., Dalke, A., Friedberg, I., Hamelryck, T., Kauff, F., Wilczynski, B., and de Hoon, M.J.L. (2009). Biopython: freely available Python tools for computational molecular biology and bioinformatics. Bioinformatics *25*, 1422–1423. https://doi.org/10.1093/bioinformatics/btp163.
75. R Core Team (2017). R: A language and environment for statistical computing. https://www.R-project.org/.
76. Rheinemann, L., Downhour, D.M., Davenport, K.A., McKeown, A.N., Sundquist, W.I., and Elde, N.C. (2022). Recurrent evolution of an inhibitor of ESCRT-dependent virus budding and LINE-1 retrotransposition in primates. Curr. Biol. *32*, 1511–1522.e6. https://doi.org/10.1016/j.cub.2022.02.018.
77. Carey, C.M., Govande, A.A., Cooper, J.M., Hartley, M.K., Kranzusch, P.J., and Elde, N.C. (2019). Recurrent Loss-of-Function Mutations Reveal Costs to OAS1 Antiviral Activity in Primates. Cell Host Microbe *25*, 336–343.e4. https://doi.org/10.1016/j.chom.2019.01.001.
78. Kozak, M. (1990). Downstream secondary structure facilitates recognition of initiator codons by eukaryotic ribosomes. Proc. Natl. Acad. Sci. USA *87*, 8301–8305. https://doi.org/10.1073/pnas.87.21.8301.
79. Fablet, M., Bueno, M., Potrzebowski, L., and Kaessmann, H. (2009). Evolutionary Origin and Functions of Retrogene Introns. Mol. Biol. Evol. *26*, 2147–2156. https://doi.org/10.1093/molbev/msp125.
80. Kazazian, H.H., and Moran, J.V. (1998). The impact of L1 retrotransposons on the human genome. Nat. Genet. *19*, 19–24. https://doi.org/10.1038/ng0598-19.
81. Aradhya, S., Bardaro, T., Galgóczy, P., Yamagata, T., Esposito, T., Patlan, H., Ciccodicola, A., Munnich, A., Kenwrick,

S., Platzer, M., et al. (2001). Multiple pathogenic and benign genomic rearrangements occur at a 35 kb duplication involving the NEMO and LAGE2 genes. Hum. Mol. Genet. *10*, 2557–2567. https://doi.org/10.1093/hmg/10.22.2557.
82. Cibelli, G., Schoch, S., and Thiel, G. (1999). Nuclear targeting of cAMP response element binding protein 2 (CREB2). Eur. J. Cell Biol. *78*, 642–649. https://doi.org/10.1016/S0171-9335(99)80049-1.
83. Bagheri-Yarmand, R., Sinha, K.M., Gururaj, A.E., Ahmed, Z., Rizvi, Y.Q., Huang, S.C., Ladbury, J.E., Bogler, O., Williams, M.D., Cote, G.J., and Gagel, R.F. (2015). A novel dual kinase function of the RET proto-oncogene negatively regulates activating transcription factor 4-mediated apoptosis. J. Biol. Chem. *290*, 11749–11761. https://doi.org/10.1074/jbc.M114.619833.
84. Shiozaki, Y., Okamura, K., Kohno, S., Keenan, A.L., Williams, K., Zhao, X., Chick, W.S., Miyazaki-Anzai, S., and Miyazaki, M. (2018). The CDK9 – cyclin T1 complex mediates saturated fatty acid–induced vascular calcification by inducing expression of the transcription factor CHOP. J. Biol. Chem. *293*, 17008–17020. https://doi.org/10.1074/jbc.RA118.004706.
85. Keightley, P.D. (2012). Rates and fitness consequences of new mutations in humans. Genetics *190*, 295–304. https://doi.org/10.1534/genetics.111.134668.
86. Yang, Y., Xu, T., Conant, G., Kishino, H., Thorne, J.L., and Ji, X. (2023). Interlocus Gene Conversion, Natural Selection, and Paralog Homogenization. Mol. Biol. Evol. *40*, msad198. https://doi.org/10.1093/molbev/msad198.
87. Gruber, T., and Clay, Z. (2016). Comparison Between Bonobos and Chimpanzees: A Review and Update. Evol. Anthropol. *25*, 239–252. https://doi.org/10.1002/evan.21501.
88. Adzhubei, I.A., Schmidt, S., Peshkin, L., Ramensky, V.E., Gerasimova, A., Bork, P., Kondrashov, A.S., and Sunyaev, S.R. (2010). A method and server for predicting damaging missense mutations. Nat. Methods *7*, 248–249. https://doi.org/10.1038/nmeth0410-248.
89. Sim, N.L., Kumar, P., Hu, J., Henikoff, S., Schneider, G., and Ng, P.C. (2012). SIFT web server: Predicting effects of amino acid substitutions on proteins. Nucleic Acids Res. *40*, W452–W457. https://doi.org/10.1093/nar/gks539.
90. Conceição, H.B., Mercuri, R.L.V., de Castro, M.P.M., Ohara, D.T., Guardia, G.D.A., and Galante, P.A.F. (2024). RCPedia: a global resource for studying and exploring retrocopies in diverse species. Bioinformatics *40*, btae530. https://doi.org/10.1093/bioinformatics/btae530.
91. Mighell, A.J., Smith, N.R., Robinson, P.A., and Markham, A.F. (2000). Vertebrate pseudogenes. FEBS Lett. *468*, 109–114. https://doi.org/10.1016/s0014-5793(00)01199-6.
92. Ciomborowska-basheer, J., Staszak, K., Kubiak, M.R., and Makałowska, I. (2021). Not so dead genes—retrocopies as regulators of their disease-related progenitors and hosts. Cells *10*, 912. https://doi.org/10.3390/cells10040912.
93. Buckley, R.M., Kortschak, R.D., and Adelson, D.L. (2018). Divergent genome evolution caused by regional variation in DNA gain and loss between human and mouse. PLoS Comput. Biol. *14*, e1006091. https://doi.org/10.1371/journal.pcbi.1006091.
94. Glinos, D.A., Garborcauskas, G., Hoffman, P., Ehsan, N., Jiang, L., Gokden, A., Dai, X., Aguet, F., Brown, K.L., Garimella, K., et al. (2022). Transcriptome variation in human tissues revealed by long-read sequencing. Nature *608*, 353–359. https://doi.org/10.1038/s41586-022-05035-y.
95. GTEx Portal. eQTL Analysis. https://www.gtexportal.org/home/methods.
96. Moreno, P., Fexova, S., George, N., Manning, J.R., Miao, Z., Mohammed, S., Muñoz-Pomer, A., Fullgrabe, A., Bi, Y., Bush, N., et al. (2022). Expression Atlas update: Gene and protein expression in multiple species. Nucleic Acids Res. *50*, D129–D140. https://doi.org/10.1093/nar/gkab1030.
97. Expression Atlas < EMBL-EBI, 'FAQ'. https://www.ebi.ac.uk/gxa/FAQ.html.
98. Liu, X.-X., Zhang, H., Shen, X.F., Liu, F.J., Liu, J., and Wang, W.J. (2016). Characteristics of testis-specific phosphoglycerate kinase 2 and its association with human sperm quality. Hum. Reprod. *31*, 273–279. https://doi.org/10.1093/humrep/dev301.
99. Sisu, C., Pei, B., Leng, J., Frankish, A., Zhang, Y., Balasubramanian, S., Harte, R., Wang, D., Rutenberg-Schoenberg, M., Clark, W., et al. (2014). Comparative analysis of pseudogenes across three phyla. Proc. Natl. Acad. Sci. USA *111*, 13361–13366. https://doi.org/10.1073/pnas.1407293111.
100. ENCODE Project Consortium (2012). An integrated encyclopedia of DNA elements in the human genome. Nature *489*, 57–74. https://doi.org/10.1038/nature11247.
101. Kubo, N., Chen, P.B., Hu, R., Ye, Z., Sasaki, H., and Ren, B. (2024). H3K4me1 facilitates promoter-enhancer interactions and gene activation during embryonic stem cell differentiation. Mol. Cell *84*, 1742–1752.e5. https://doi.org/10.1016/j.molcel.2024.02.030.
102. Creyghton, M.P., Cheng, A.W., Welstead, G.G., Kooistra, T., Carey, B.W., Steine, E.J., Hanna, J., Lodato, M.A., Frampton, G.M., Sharp, P.A., et al. (2010). Histone H3K27ac separates active from poised enhancers and predicts developmental state. Proc. Natl. Acad. Sci. USA *107*, 21931–21936. https://doi.org/10.1073/pnas.1016071107.
103. Lauberth, S.M., Nakayama, T., Wu, X., Ferris, A.L., Tang, Z., Hughes, S.H., and Roeder, R.G. (2013). H3K4me3 interactions with TAF3 regulate preinitiation complex assembly and selective gene activation. Cell *152*, 1021–1036. https://doi.org/10.1016/j.cell.2013.01.052.
104. Badu, P., Baniulyte, G., Sammons, M.A., and Pager, C.T. (2024). Activation of ATF3 via the integrated stress response pathway regulates innate immune response to restrict Zika virus. J. Virol. *98*, e0105524. https://doi.org/10.1128/jvi.01055-24.
105. Jiang, H.-Y., Wek, S.A., McGrath, B.C., Lu, D., Hai, T., Harding, H.P., Wang, X., Ron, D., Cavener, D.R., and Wek, R.C. (2004). Activating Transcription Factor 3 Is Integral to the Eukaryotic Initiation Factor 2 Kinase Stress Response. Mol. Cell Biol. *24*, 1365–1377. https://doi.org/10.1128/MCB.24.3.1365-1377.2004.
106. Kalinin, A., Zubkova, E., and Menshikov, M. (2023). Integrated Stress Response (ISR) Pathway: Unraveling Its Role in Cellular Senescence. Int. J. Mol. Sci. *24*, 17423. https://doi.org/10.3390/ijms242417423.
107. hTERT RPE-1 - CRL-4000 | ATCC. https://www.atcc.org/products/crl-4000.
108. Hindul, N.L., Jhita, A., Oprea, D.G., Hussain, T.A., Gonchar, O., Campillo, M.A.M., O'Regan, L., Kanemaki, M.T., Fry, A.M., Hirota, K., and Tanaka, K. (2022). Construction of a human hTERT RPE-1 cell line with inducible Cre for editing of endogenous genes. Biol. Open *11*, bio059056. https://doi.org/10.1242/bio.059056.

109. Taniuchi, S., Miyake, M., Tsugawa, K., Oyadomari, M., and Oyadomari, S. (2016). Integrated stress response of vertebrates is regulated by four eIF2α kinases. Sci. Rep. *6*, 32886. https://doi.org/10.1038/srep32886.
110. Armstrong, J.L., Flockhart, R., Veal, G.J., Lovat, P.E., and Redfern, C.P.F. (2010). Regulation of endoplasmic reticulum stress-induced cell death by ATF4 in neuroectodermal tumor cells. J. Biol. Chem. *285*, 6091–6100. https://doi.org/10.1074/jbc.M109.014092.
111. Dey, S., Baird, T.D., Zhou, D., Palam, L.R., Spandau, D.F., and Wek, R.C. (2010). Both transcriptional regulation and translational control of ATF4 are central to the integrated stress response. J. Biol. Chem. *285*, 33165–33174. https://doi.org/10.1074/jbc.M110.167213.
112. Wakabayashi, Y., Shimono, A., Terauchi, Y., Zeng, C., Hamada, M., Semba, K., Watanabe, S., and Ishikawa, K. (2024). Identification of a novel RNA transcript TISPL upregulated by stressors that stimulate ATF4. Gene *917*, 148464. https://doi.org/10.1016/j.gene.2024.148464.
113. Lu, L., Han, A.-P., and Chen, J.-J. (2001). Translation Initiation Control by Heme-Regulated Eukaryotic Initiation Factor 2α Kinase in Erythroid Cells under Cytoplasmic Stresses. Mol. Cell Biol. *21*, 7971–7980. https://doi.org/10.1128/MCB.21.23.7971-7980.2001.
114. Örd, T., Örd, D., Kaikkonen, M.U., and Örd, T. (2021). Pharmacological or trib3-mediated suppression of atf4 transcriptional activity promotes hepatoma cell resistance to proteasome inhibitor bortezomib. Cancers (Basel) *13*, 2341. https://doi.org/10.3390/cancers13102341.
115. Nunes, A.T., and Annunziata, C.M. (2017). Proteasome inhibitors: structure and function. Semin. Oncol. *44*, 377–380. https://doi.org/10.1053/j.seminoncol.2018.01.004.
116. Ruschak, A.M., Religa, T.L., Breuer, S., Witt, S., and Kay, L.E. (2010). The proteasome antechamber maintains substrates in an unfolded state. Nature *467*, 868–871. https://doi.org/10.1038/nature09444.
117. Navarro, F.C.P., and Galante, P.A.F. (2015). A Genome-Wide Landscape of Retrocopies in Primate Genomes. Genome Biol. Evol. *7*, 2265–2275. https://doi.org/10.1093/gbe/evv142.
118. Yan, Y., Tian, Y., Wu, Z., Zhang, K., and Yang, R. (2023). Interchromosomal Colocalization with Parental Genes Is Linked to the Function and Evolution of Mammalian Retrocopies. Mol. Biol. Evol. *40*, msad265. https://doi.org/10.1093/molbev/msad265.
119. Chen, M., Liu, Y., Yang, Y., Qiu, Y., Wang, Z., Li, X., and Zhang, W. (2022). Emerging roles of activating transcription factor (ATF) family members in tumourigenesis and immunity: Implications in cancer immunotherapy. Genes Dis. *9*, 981–999. https://doi.org/10.1016/j.gendis.2021.04.008.
120. Kovács, D., Kovács, M., Ahmed, S., and Barna, J. (2022). Functional diversification of heat shock factors. Biol. Futur. *73*, 427–439. https://doi.org/10.1007/s42977-022-00138-z.
121. Rosikiewicz, W., Kabza, M., Kosinski, J.G., Ciomborowska-Basheer, J., Kubiak, M.R., and Makalowska, I. (2017). RetrogeneDB–a database of plant and animal retrocopies. Database. *2017*, bax038. https://doi.org/10.1093/database/bax038.
122. Lonsdale, J., Thomas, J., Salvatore, M., Phillips, R., Lo, E., Shad, S., Hasz, R., Walters, G., Garcia, F., Young, N., et al. (2013). The Genotype-Tissue Expression (GTEx) project. Nat. Genet. *45*, 580–585. https://doi.org/10.1038/ng.2653.
123. Castilho, B.A., Shanmugam, R., Silva, R.C., Ramesh, R., Himme, B.M., and Sattlegger, E. (2014). Keeping the eIF2 alpha kinase Gcn2 in check. Biochim. Biophys. Acta *1843*, 1948–1968. https://doi.org/10.1016/j.bbamcr.2014.04.006.
124. Delépine, M., Nicolino, M., Barrett, T., Golamaully, M., Lathrop, G.M., and Julier, C. (2000). EIF2AK3, encoding translation initiation factor 2-α kinase 3, is mutated in patients with Wolcott-Rallison syndrome. Nat. Genet. *25*, 406–409. https://doi.org/10.1038/78085.
125. Emanuelli, G., Nassehzadeh-Tabriz, N., Morrell, N.W., and Marciniak, S.J. (2020). The integrated stress response in pulmonary disease. Eur. Respir. Rev. *29*, 200184. https://doi.org/10.1183/16000617.0184-2020.
126. Yang, W., Zhou, X., Zimmermann, H.R., Cavener, D.R., Klann, E., and Ma, T. (2016). Repression of the eIF2α kinase PERK alleviates mGluR-LTD impairments in a mouse model of Alzheimer's disease. Neurobiol. Aging *41*, 19–24. https://doi.org/10.1016/j.neurobiolaging.2016.02.005.
127. Dey, S., Sayers, C.M., Verginadis, I.I., Lehman, S.L., Cheng, Y., Cerniglia, G.J., Tuttle, S.W., Feldman, M.D., Zhang, P.J.L., Fuchs, S.Y., et al. (2015). ATF4-dependent induction of heme oxygenase 1 prevents anoikis and promotes metastasis. J. Clin. Investig. *125*, 2592–2608. https://doi.org/10.1172/JCI78031.
128. Lin, Y., Pang, X., Huang, G., Jamison, S., Fang, J., Harding, H.P., Ron, D., and Lin, W. (2014). Impaired eukaryotic translation initiation factor 2B activity specifically in oligodendrocytes reproduces the pathology of vanishing white matter disease in mice. J. Neurosci. *34*, 12182–12191. https://doi.org/10.1523/JNEUROSCI.1373-14.2014.
129. Lin, J.H., Li, H., Yasumura, D., Cohen, H.R., Zhang, C., Panning, B., Shokat, K.M., Lavail, M.M., and Walter, P. (2007). IRE1 signaling affects cell fate during the unfolded protein response. Science *318*, 944–949. https://doi.org/10.1126/science.1146361.
130. Matz, A., Lee, S.J., Schwedhelm-Domeyer, N., Zanini, D., Holubowska, A., Kannan, M., Farnworth, M., Jahn, O., Göpfert, M.C., and Stegmüller, J. (2015). Regulation of neuronal survival and morphology by the E3 ubiquitin ligase RNF157. Cell Death Differ. *22*, 626–642. https://doi.org/10.1038/cdd.2014.163.
131. Steger, M., Demichev, V., Backman, M., Ohmayer, U., Ihmor, P., Müller, S., Ralser, M., and Daub, H. (2021). Time-resolved in vivo ubiquitinome profiling by DIA-MS reveals USP7 targets on a proteome-wide scale. Nat. Commun. *12*, 5399. https://doi.org/10.1038/s41467-021-25454-1.
132. Hottiger, M.O., and Nabel, G.J. (2000). Viral replication and the coactivators p300 and CBP. Trends Microbiol. *8*, 560–565. https://doi.org/10.1016/s0966-842x(00)01874-6.
133. D'Orso, I., and Frankel, A.D. (2009). Tat acetylation modulates assembly of a viral-host RNA-protein transcription complex. Proc. Natl. Acad. Sci. USA *106*, 3101–3106. https://doi.org/10.1073/pnas.0900012106.
134. Bostik, P., Noble, E.S., Stephenson, S.T., Villinger, F., and Ansari, A.A. (2007). CD4+ T Cells from Simian Immunodeficiency Virus Disease-Resistant Sooty Mangabeys Produce More IL-2 Than Cells from Disease-Susceptible Species: Involvement of p300 and CREB at the Proximal IL-2 Promoter in IL-2 Up-Regulation. J. Immunol. *178*, 7720–7729. https://doi.org/10.4049/jimmunol.178.12.7720.
135. Ott, M., Schnölzer, M., Garnica, J., Fischle, W., Emiliani, S., Rackwitz, H.R., and Verdin, E. (1999). Acetylation of the HIV-1 Tat protein by p300 is important for its

transcriptional activity. Curr. Biol. *9*, 1489–1492. https://doi.org/10.1016/s0960-9822(00)80120-7.

136. Ferrari, R., Gou, D., Jawdekar, G., Johnson, S.A., Nava, M., Su, T., Yousef, A.F., Zemke, N.R., Pellegrini, M., Kurdistani, S.K., and Berk, A.J. (2014). Adenovirus small E1A employs the lysine acetylases p300/CBP and tumor suppressor RB to repress select host genes and promote productive virus infection. Cell Host Microbe *16*, 663–676. https://doi.org/10.1016/j.chom.2014.10.004.

137. Duvaud, S., Gabella, C., Lisacek, F., Stockinger, H., Ioannidis, V., and Durinx, C. (2021). Expasy, the Swiss Bioinformatics Resource Portal, as designed by its users. Nucleic Acids Res. *49*, W216–W227. https://doi.org/10.1093/nar/gkab225.

138. Perez, G., Barber, G.P., Benet-Pages, A., Casper, J., Clawson, H., Diekhans, M., Fischer, C., Gonzalez, J.N., Hinrichs, A.S., Lee, C.M., et al. (2025). The UCSC Genome Browser database: 2025 update. Nucleic Acids Res. *53*, D1243–D1249. https://doi.org/10.1093/nar/gkae974.

HGGA, Volume *7*

Supplemental information

The stress-induced transcription factor ATF4 has multiple conserved retrocopies that can alter gene expression

Hans M. Dalton, Elizabeth M. Brydon, Tiffany S. Chan, Katie G. Owings, Mandi M.L. Wild, Naomi J. Young, and Clement Y. Chow

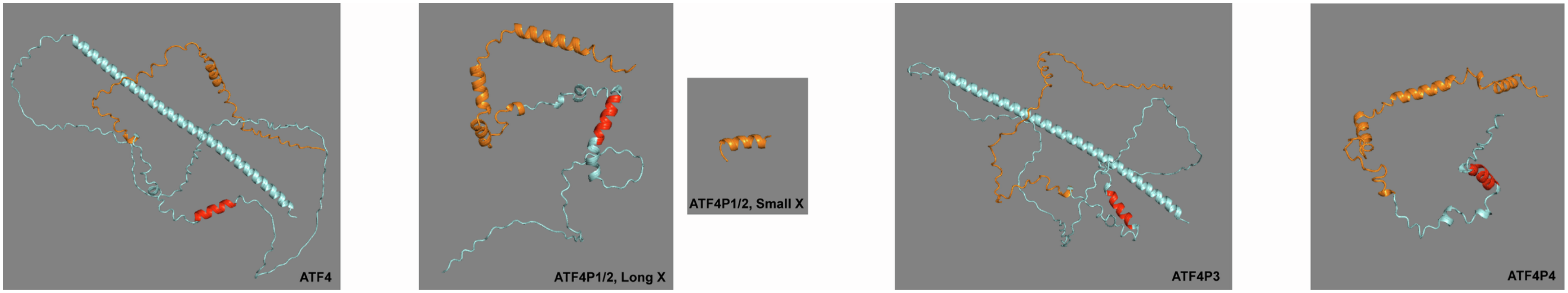

Figure S1. AlphaFold predictions of ATF4 retrocopy protein structures. Alphafold 3 predictions of translated retrocopy transcripts, visualized in PyMOL. Specific protein domains are colored for comparison: the p300 interaction domain (orange), an alpha helix structure that is maintained in all proteins except ATF4P1/2 Small X (red), and the rest of the protein including the basic leucine zipper domain (blue

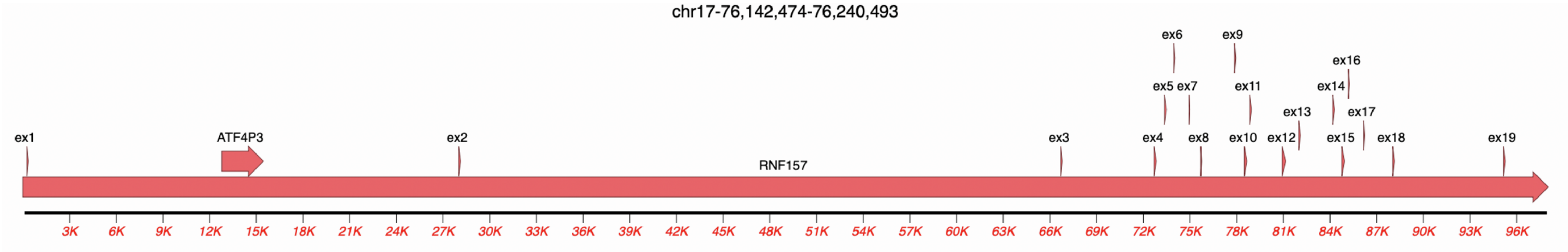

Figure S2. Mock-up of the genomic region containing the gene *RNF157*. *ATF4P3* was inserted between exons 1 and 2. Image was created using MacVector™ v.18.2.5.

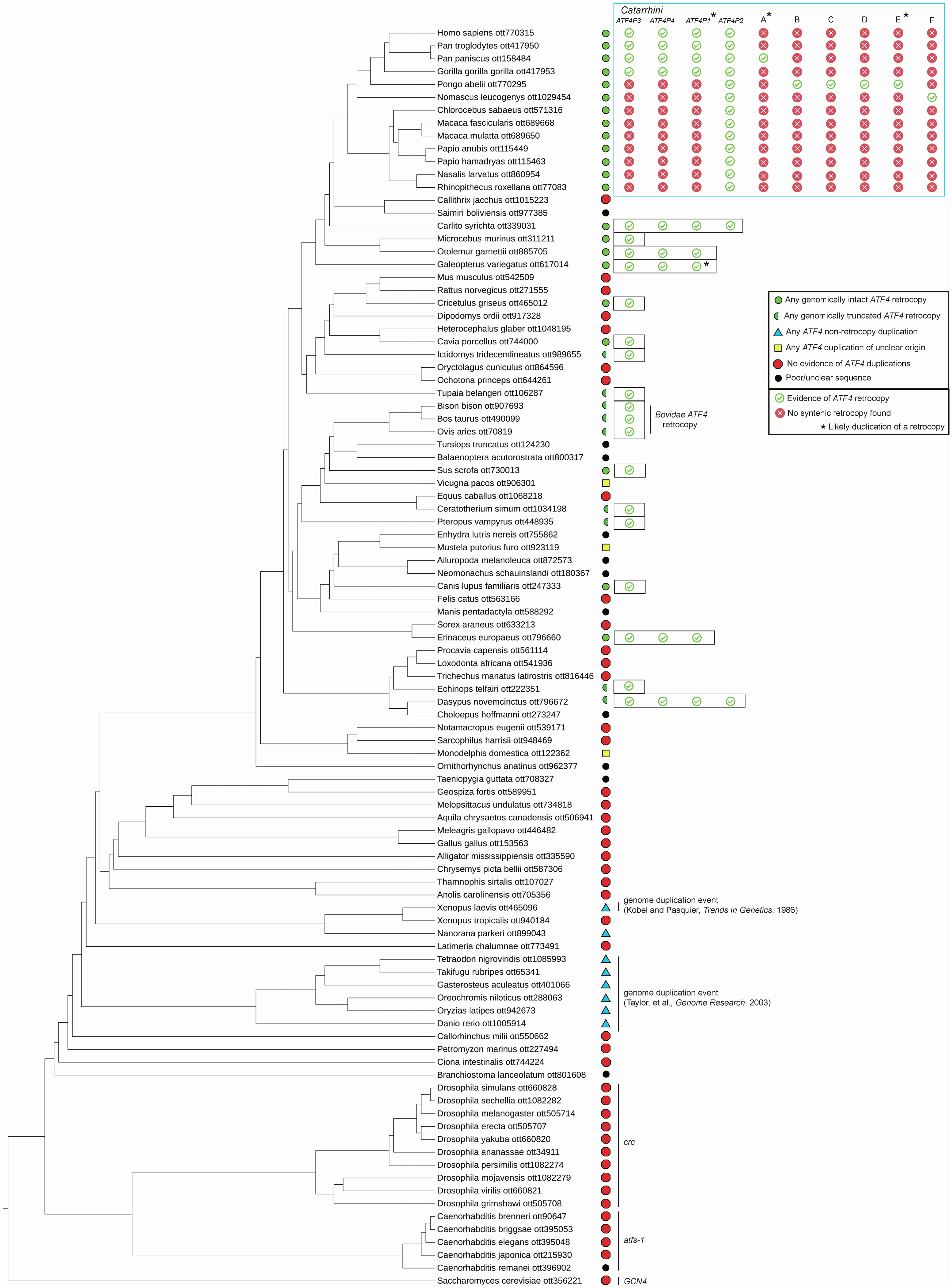

Catarrhini
ATF4P3
ATF4P4
ATF4P1*
ATF4P2
A*
B
C
D
E*
F
Homo sapiens ott770315
Pan troglodytes ott417950
Pan paniscus ott158484
Gorilla gorilla gorilla ott417953
Pongo abelii ott770295
Nomascus leucogenys ott1029454
Chlorocebus sabaeus ott571316
Macaca fascicularis ott689668
Macaca mulatta ott689650
Papio anubis ott115449
Papio hamadryas ott115463
Nasalis larvatus ott860954
Rhinopithecus roxellana ott77083
Callithrix jacchus ott1015223
Saimiri boliviensis ott977385
Carlito syrichta ott339031
Microcebus murinus ott311211
Otolemur garnettii ott885705
Galeopterus variegatus ott617014
Mus musculus ott542509
Rattus norvegicus ott271555
Cricetulus griseus ott465012
Dipodomys ordii ott917328
Heterocephalus glaber ott1048195
Cavia porcellus ott744000
Ictidomys tridecemlineatus ott989655
Oryctolagus cuniculus ott864596
Ochotona princeps ott644261
Tupaia belangeri ott106287
Bison bison ott907693
Bos taurus ott490099
Ovis aries ott70819
Bovidae ATF4 retrocopy
Tursiops truncatus ott124230
Balaenoptera acutorostrata ott800317
Sus scrofa ott730013
Vicugna pacos ott906301
Equus caballus ott1068218
Ceratotherium simum ott1034198
Pteropus vampyrus ott448935
Enhydra lutris nereis ott755862
Mustela putorius furo ott923119
Ailuropoda melanoleuca ott872573
Neomonachus schauinslandi ott180367
Canis lupus familiaris ott247333
Felis catus ott563166
Manis pentadactyla ott588292
Sorex araneus ott633213
Erinaceus europaeus ott796660
Procavia capensis ott561114
Loxodonta africana ott541936
Trichechus manatus latirostris ott816446
Echinops telfairi ott222351
Dasypus novemcinctus ott796672
Choloepus hoffmanni ott273247
Notamacropus eugenii ott539171
Sarcophilus harrisii ott948469
Monodelphis domestica ott122362
Ornithorhynchus anatinus ott962377
Taeniopygia guttata ott708327
Geospiza fortis ott589951
Melopsittacus undulatus ott734818
Aquila chrysaetos canadensis ott506941
Meleagris gallopavo ott446482
Gallus gallus ott153563
Alligator mississippiensis ott335590
Chrysemys picta bellii ott587306
Thamnophis sirtalis ott107027
Anolis carolinensis ott705356
Xenopus laevis ott465096
Xenopus tropicalis ott940184
Nanorana parkeri ott899043
genome duplication event (Kobel and Pasquier, Trends in Genetics, 1986)
Latimeria chalumnae ott773491
Tetraodon nigroviridis ott1085993
Takifugu rubripes ott65341
Gasterosteus aculeatus ott401066
Oreochromis niloticus ott288063
Oryzias latipes ott942673
Danio rerio ott1005914
genome duplication event (Taylor, et al., Genome Research, 2003)
Callorhinchus milii ott550662
Petromyzon marinus ott227494
Ciona intestinalis ott744224
Branchiostoma lanceolatum ott801608
Drosophila simulans ott660828
Drosophila sechellia ott1082282
Drosophila melanogaster ott505714
Drosophila erecta ott505707
Drosophila yakuba ott660820
Drosophila ananassae ott34911
Drosophila persimilis ott1082274
Drosophila mojavensis ott1082279
Drosophila virilis ott660821
Drosophila grimshawi ott505708
crc
Caenorhabditis brenneri ott90647
Caenorhabditis briggsae ott395053
Caenorhabditis elegans ott395048
Caenorhabditis japonica ott215930
Caenorhabditis remanei ott396902
atfs-1
Saccharomyces cerevisiae ott356221
GCN4
Any genomically intact ATF4 retrocopy
Any genomically truncated ATF4 retrocopy
Any ATF4 non-retrocopy duplication
Any ATF4 duplication of unclear origin
No evidence of ATF4 duplications
Poor/unclear sequence
Evidence of ATF4 retrocopy
No syntenic retrocopy found
* Likely duplication of a retrocopy

Figure S3. Species tree of all organisms that we queried for *ATF4* retrocopies. Various colored shapes represent the status of any *ATF4* retrocopy and/or duplication found upon searching BLAT (with a BLAT score of at least 100). *Catarrhini* retrocopies, containing all retrocopies of *ATF4* found in humans, are contained in a blue box. In this box, checkmarks represent the presence of an *ATF4* retrocopy being found syntenic to *ATF4P1-P4*, or independent *ATF4* retrocopies that are neither syntenic nor duplications of *ATF4P1-P4* (letters A-F). Outside of this box, each horizontal box represents independent retrocopies of *ATF4* (not related to *ATF4P1-4*), while the single vertical box (labeled *Bovidae* retrocopy) is another syntenic retrocopy of *ATF4*. For example, for *Carlito syrichta*, it has four independent retrocopies unique to itself (i.e. these are not *ATF4P1-4* and are not syntenic to any other primate *ATF4* retrocopy). We also note two cases where entire genomes were duplicated (and *ATF4* with them) in *Xenopus*[1] and ray-finned fish[2]. Finally, we note that we searched the listed closest invertebrate homologs in *Drosophila*, *Caenorhabditis*, and *S. cerevisiae*, none of which had retrocopies.

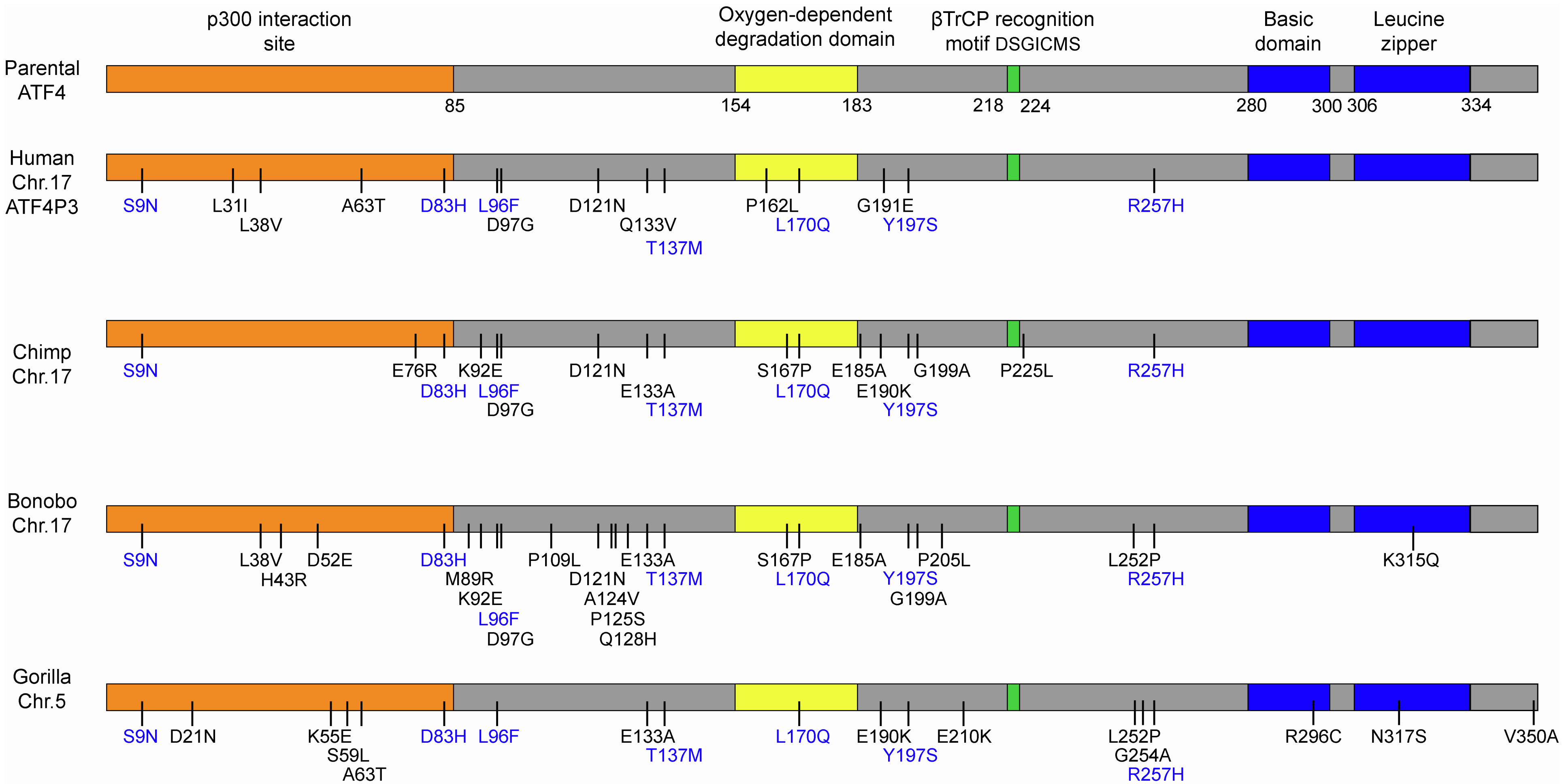

Figure S4. Mock-up of ATF4P3 across humans, chimps, bonobos, and gorillas. We compared protein sequences to the species-specific ATF4 parent gene to determine amino acid changes. Amino acid changes that are shared across all four species are labeled in blue.

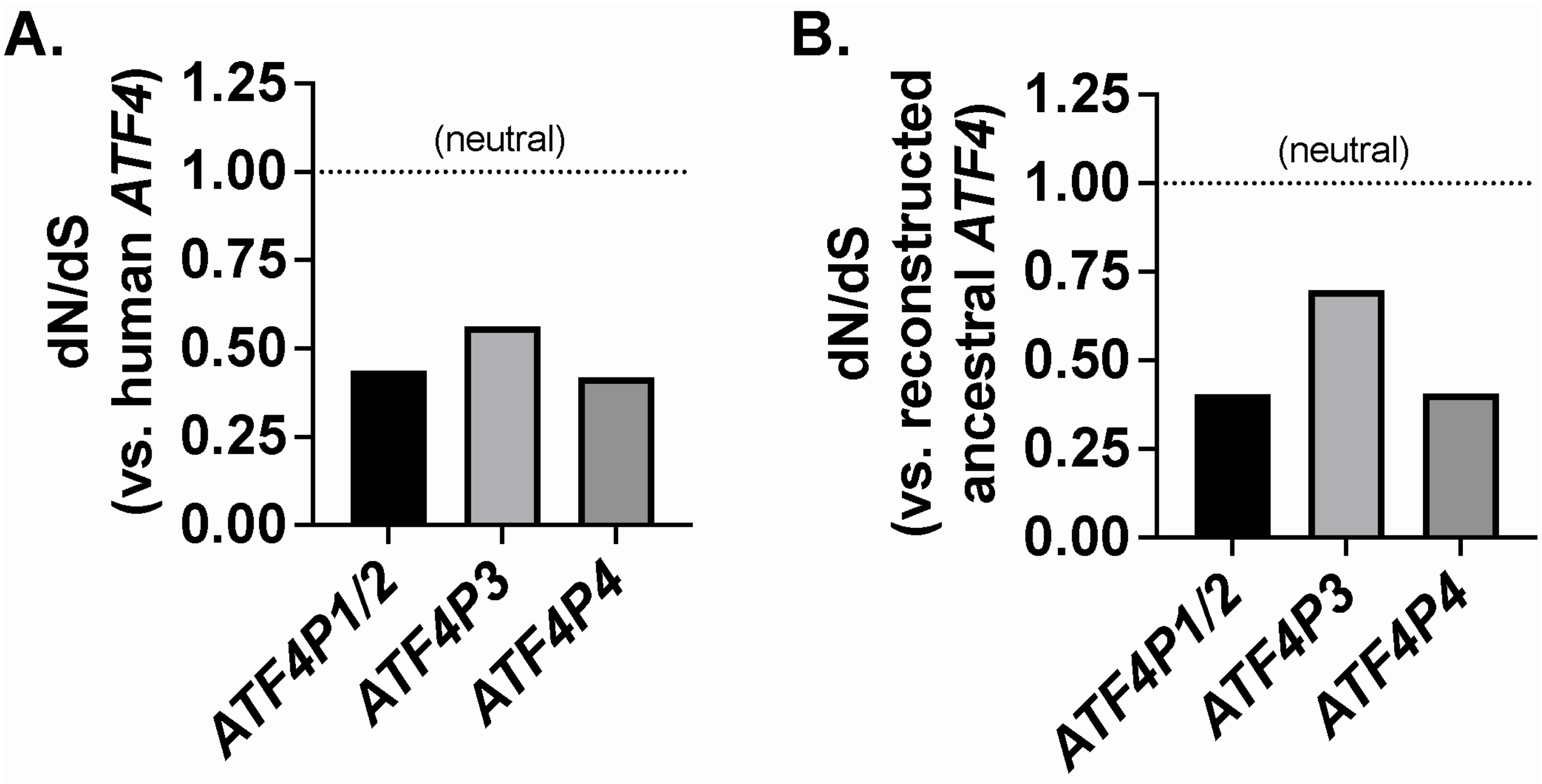

Figure S5. Graphs of dN/dS calculations on *ATF4* retrocopies. A. Graph showing calculated dN/dS of *ATF4* retrocopies compared to the human *ATF4* parent gene. B. Graph showing calculated dN/dS of *ATF4* retrocopies compared to the ancestral reconstructions of *ATF4* (Table S3). The dotted line at 1.0 indicates neutral evolution, while below line represents negative selection.

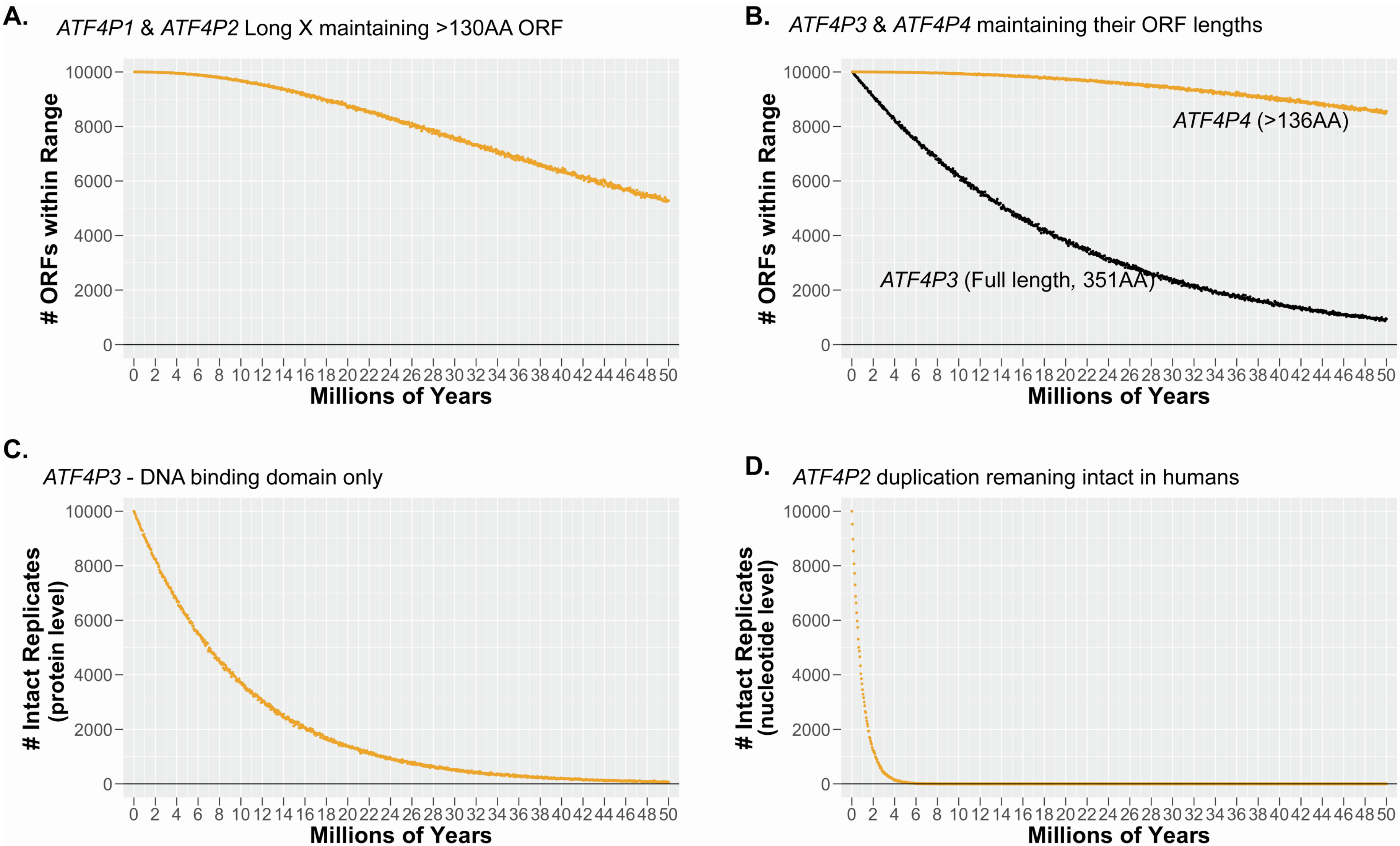

Figure S6. Graphs of computationally modeled mutation rates of *ATF4* retrocopies. A. Graph showing how many permutations (out of 10,000) of our estimated ancestral copy of *ATF4P1/P2* maintaining a >130 AA ORF over time. B. Same as (A), but for ATF4P3 and ATF4P4 maintaining either >136 AA ORF (*ATFP4*) or a full length ORF (*ATF4P3*). C. Same as (A), but for ATF4P4 maintaining its DNA binding domain fully intact. D. Same as

(A), but for *ATF4P2* duplication remaining invariant. These graphs were used to determine probabilities of intactness across species - see Methods for further details.

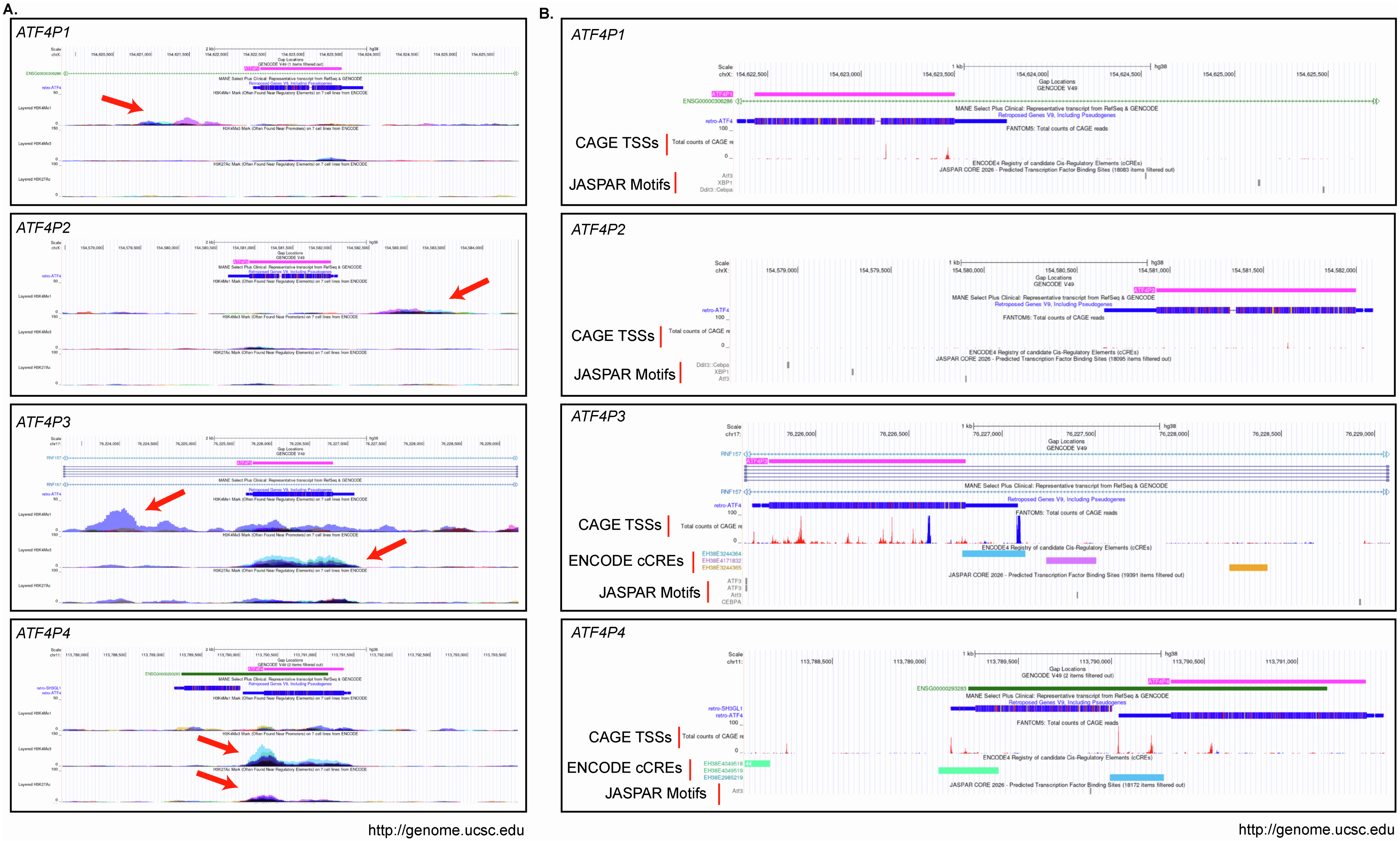

Figure S7. UCSC genome browser navigated to retrocopies with tracks for regulatory information. A. Tracks containing the chromatin markers H3K27Ac, H3K4Me1, and H3K4Me3 are shown within a 6 kb window. We point out peaks that are higher than surrounding areas with red arrows. Note that >2 kb on either side display no H3K4Me3 marks, suggesting specificity to each retrocopy. B. Tracks containing cCREs from ENCODE,

transcription start site (TSS) peaks from CAGE data, and any JASPAR motifs for ISR/UPR-specific transcription factors (see Methods) are shown. For cCREs: blue = high chromatin accessibility/CTCF signals, but low H3K4me3/H3K27ac signals; mauve = low chromatin accessibility/chromatin markers/CTCF signals, but are bound by a transcription factor; orange = high chromatin accessibility/H3K27ac signals and within 2 kb of a TSS; and green = high chromatin accessibility, but low H3K4me3/H3K27ac/CTCF signals. Note that the cCRE track is on for *ATF4P1* and *ATF4P2*, but no cCREs are present.

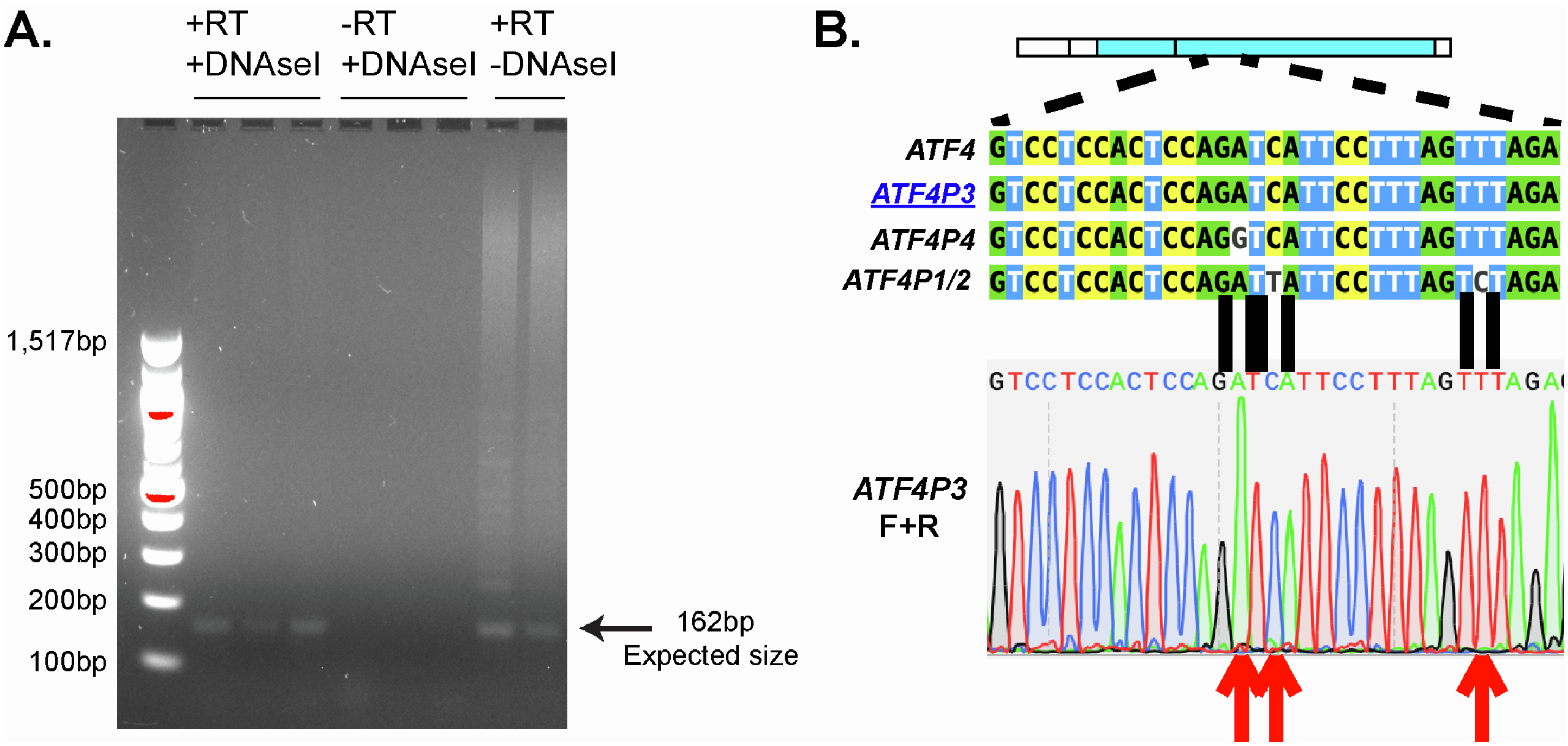

Figure S8. DNAse efficacy and primer specificity. A. Agarose gel showing the effectiveness of the Zymo DirectZol™ kit at removing genomic DNA from our TriZol™-derived RNA samples of RPE-1 human cells. We used the *ATF4* Forward and Reverse primers listed for qPCR. Lane 1 is a 100 bp ladder (NEB, cat. N3231L) (the two red splotches represent hitting the light threshold for two sizes that are unrelated to the results). Lanes 2-4 are three biological replicates using the DirectZol kit with the optional DNAseI treatment. Lanes 5-7 are three biological replicates the same procedure but omitting the reverse transcriptase reagent. Lanes 8-9 are biological replicates using the DirectZol kit without the DNAseI treatment. If our prepped RNA sample contained residual genomic DNA, that the DNAseI treatment did not destroy, we would have seen bands in lanes 5-7. Thus, we conclude that the DNAseI treatment does fully remove residual genomic DNA, and that lanes 2-4 (and the data in Figure 3) are derived from actual expressed mRNA. Lanes 8-9 are for reference only to show that DNAse I does have an effect on removing DNA. B. Additional view of chromatogram of sequenced DNA product using ATF4P3 primers. This plot shows three additional SNPs, one for *ATF4P4* and two for *ATF4P1/2*, each of which does not show contamination of the *ATF4P3* transcript.

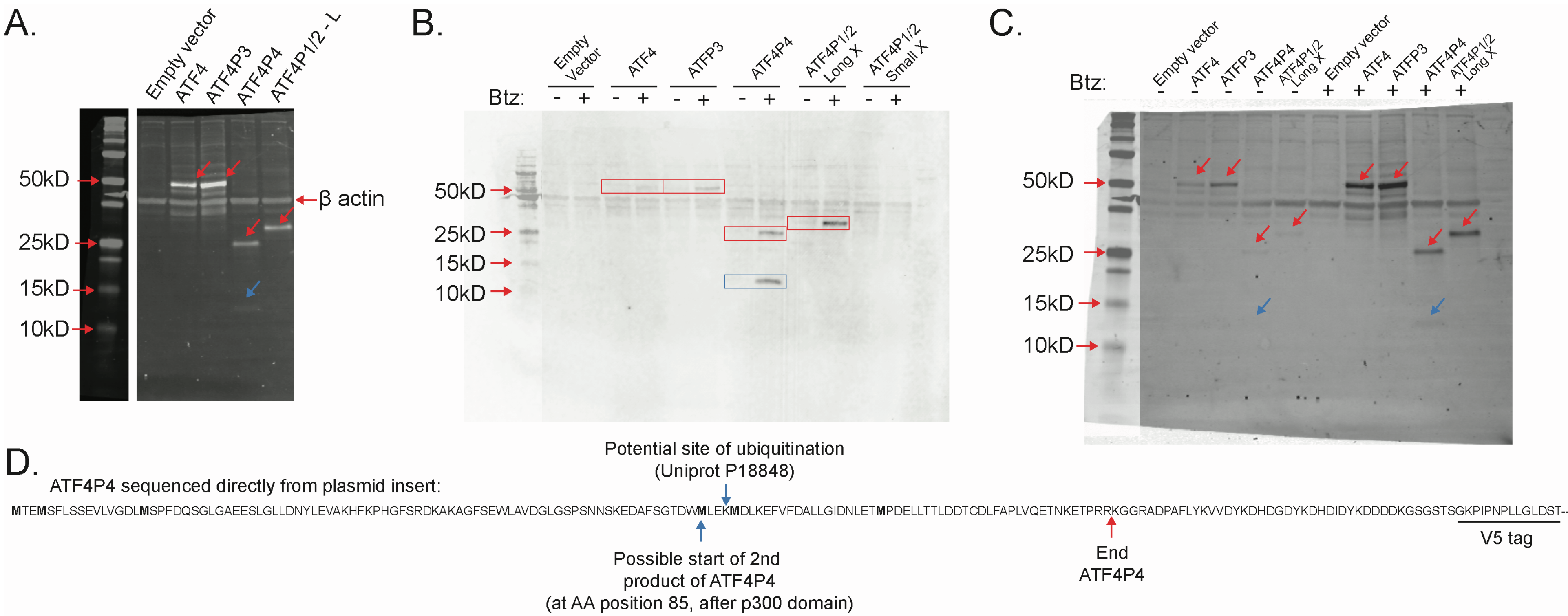

Figure S9. Images and descriptions of raw Western blots. A. Western blot of protein extracts from each OE ATF4 cell line using the anti-V5 antibody with no treatment(s). B. Uncropped Western blot of Figure 4. We note here that Btz treatment did not upregulate a visible band for the ATF4P1/2 Small X OE cell line. C. A second biological replicate of a Btz-treatment Western blot, as in (B) and Figure 4. This blot was globally contrast-adjusted to visualize fainter bands. D. Sequenced plasmid insert of ATF4P4 overexpression clone, starting at the ATF4P4 ORF. Note that the ladders are part of the original blot (and are overlaid in their general position on the blot) but are imaged using a different fluorophore, hence the difference in contrast in the ladder.

Supplemental references.

1. Kobel, H. R. & Du Pasquier, L. Genetics of polyploid Xenopus. *Trends Genet.* **2**, (1986).
2. Taylor, J. S. *et al.* Genome duplication, a trait shared by 22,000 species of ray-finned fish. *Genome Res.* **13**, (2003).
